# Dynamic O-GlcNAcylation coordinates etoposide-triggered tumor cell pyroptosis by regulating p53 stability

**DOI:** 10.1016/j.jbc.2024.108050

**Published:** 2024-12-10

**Authors:** Jing Wang, Yida Wang, Huan Xiao, Wanyi Yang, Weibo Zuo, Ziming You, Chuanfang Wu, Jinku Bao

**Affiliations:** 1 Key Laboratory of Bio-Resource and Eco-Environment of Ministry of Education, College of Life Science, Sichuan University, Chengdu, China; 2 State Key Laboratory of Oral Diseases, West China Hospital of Stomatology, Sichuan University, Chengdu, Sichuan, China

**Keywords:** O-GlcNAcylation, pyroptosis, p53, ubiquitination, MDM2

## Abstract

O-GlcNAcylation, a modification of nucleocytoplasmic proteins in mammals, plays a critical role in various cellular processes. However, the interplay and their underlying mechanisms in chemotherapy-induced tumor regression between O-GlcNAcylation and pyroptosis, a form of programmed cell death associated with innate immunity, remains unclear. Here, we observed that during the etoposide-induced pyroptosis of SH-SY5Y and A549 cells, overall O-GlcNAcylation levels are substantially reduced. Pharmacological inhibition or genetic manipulation of O-GlcNAcylation, such as OGT inhibition or OGA overexpression, sensitized these cells to etoposide-induced pyroptosis both *in vitro* and *in vivo*. Mechanistically, mutations at S96 and S149 residues attenuated p53 O-GlcNAcylation, weakening its interaction with MDM2, reducing p53 ubiquitination, and increasing protein stability. These results suggest that S96 may be a putative O-GlcNAcylation site. Therefore, p53 target genes—*Fas*, *DR-5*, *Puma*, and *PIDD*—were transcriptionally upregulated, leading to activation of the caspase-3—GSDME axis and promoting etoposide-induced pyroptosis in various tumor cells. This study demonstrates a previously uncharacterized association between O-GlcNAcylation and chemotherapy-induced pyroptosis, offering potential therapeutic interventions for pyroptosis-related diseases.

Pyroptosis, a programmed cell death mediated by the Gasdermin (GSDM) family (1, 2) of proteins, has become a therapeutic target for various diseases, including cancer (3), infectious diseases (4), metabolic disorders (5), and neural-related diseases (6). Pyroptosis can be triggered by internal or external factors, such as inflammation, reactive oxygen species, chemicals, and toxins, thereby leading to the loss of cell membrane integrity (7) and the initiation of inflammation (8). Due to its unique biological characteristics, pyroptosis is emerging as a promising focus in tumor research, with potential applications in inhibiting tumor cell proliferation (9, 10, 11, 12), migration (13), and invasion (14). Moreover, pyroptosis can overcome the apoptotic resistance of tumor cells (15) and enhance antitumor immunity (16, 17). Shao *et al.* discovered that chemotherapy-induced tumor pyroptosis occurs *via* caspase-3-dependent GSDME activation (18, 19), though the regulatory mechanisms remain unclear.

Etoposide, a chemotherapeutic agent, functions as a type II topoisomerase inhibitor (20) and is widely used for treating lymphomas, lung cancer, and certain pediatric tumors such as neuroblastoma (21). Its cytotoxicity is primarily attributed to DNA damage induction (22). P53 plays a central role in the DNA damage response (DDR), determining cell fate by regulating cell cycle arrest and apoptosis (23). The DDR kinases, including ATM or ATR, phosphorylate p53 through checkpoint kinases CHK2 and CHK1, respectively, triggering p53 activation. Tetrameric p53 binds specific promoter sites to induce the transcription of downstream target genes such as *p21*, *Puma*, and *Bax*, thus mediating cellular responses (24). Post-translational modifications regulate the transcriptional activity of p53 and modulate its DNA-binding capacity during the DDR. Previous studies have associated decreased GSDME mRNA levels with etoposide resistance (25). In recent years, GSDME has been identified as the execution molecule of pyroptosis, indicating a potential relationship between etoposide treatment and pyroptosis induction.

O-linked N-acetylglucosamine (O-GlcNAc) is a dynamic monosaccharide modification of various nucleocytoplasmic proteins in mammals (26, 27). The sugar donor of this modification, uridine 5-diphospho-N-acetylglucosamine (UDP-GlcNAc), originates from the hexosamine biosynthetic pathway (HBP) (28). UDP-GlcNAc transfers O-linked β-N-acetylglucosamine (O-GlcNAc) to O-GlcNAc transferase (OGT), which adds O-GlcNAc to serine and/or threonine residues of substrate proteins, while O-GlcNAcase (OGA) removes it from the proteins (29). O-GlcNAc-modified proteins affect essential functions, such as protein-protein interactions, altering protein stability and activity (30). It has been observed that cells rapidly alter their O-GlcNAcylation levels in response to stress, with O-GlcNAcylation protecting against cellular stress and cell death, including apoptosis (31), autophagy (32), and ferroptosis (33). However, whether O-GlcNAcylation also responds to pro-pyroptotic stress remains unclear.

Here, we demonstrate that O-GlcNAcylation senses the pyroptotic stress and regulates p53 stability during pyroptosis. Our findings reveal that p53 stability initially increases during etoposide-induced pyroptosis, associated with reduced O-GlcNAcylation. De-O-GlcNAcylation of the p53 at S96 and S149 increases the transcriptional upregulation of *Fas*, *DR-5*, *Puma*, and *PIDD*, leading to activation of the caspase-3－GSDME pathway and promoting pyroptosis. These findings may uncover novel regulatory mechanisms and identify potential targets for treating pyroptosis-related diseases.

## Results

### Decrease in O-GlcNAcylation during etoposide-induced pyroptosis

**Figure 1:**
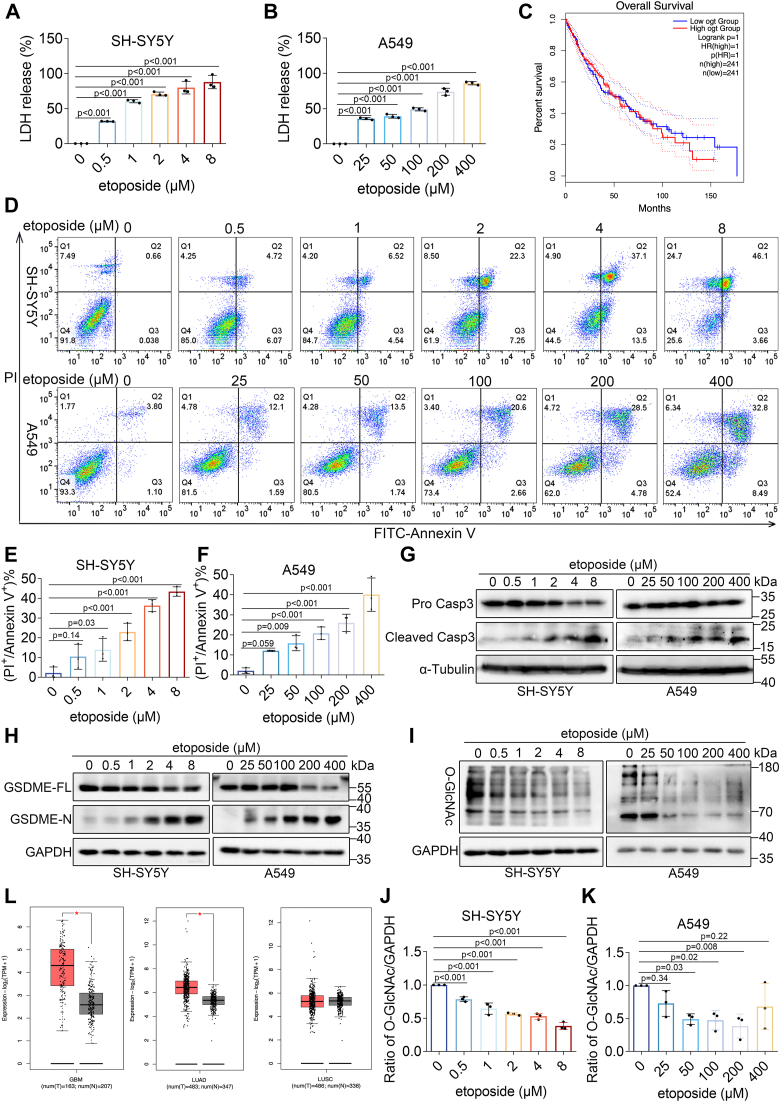
**Decrease in O-GlcNAcylation during etoposide-induced pyroptosis.***A* and *B*, LDH release in SH-SY5Y (*A*) and A549 cells (*B*) treated with different concentrations of etoposide. *C*, relationship between OGT expression and patient survival prognosis. *D*, flow cytometry analysis of Annexin V and PI staining in SH-SY5Y and A549 cells treated with different etoposide concentrations shows the percentage of double-positive cells. *E* and *F*, quantification of the percentage of double-positive cells from panel (*D*). *G*–*I*, Western blot analysis of caspase-3 (*G*), GSDME (*H*), and O-GlcNAc (*I*) in SH-SY5Y and A549 cells treated with etoposide. *J*–*K*, relative expression levels of O-GlcNAcylation in SH-SY5Y and A549 cells. *L*, mRNA expression of OGA in tumor *versus* standard samples from the TCGA database. Quantification is presented as mean ± SD (n = 3), with significance determined by one-way ANOVA (*p* < 0.05 considered statistically significant in this and subsequent figures).

Previous studies have demonstrated that chemotherapy can induce pyroptosis through the cleavage of gasdermin E (GSDME), although many tumor cells show low or no expression of GSDME due to promoter methylation (34). Several cancer cell lines were screened for GSDME expression, and SH-SY5Y and A549 cell lines were selected due to their GSDME expression and sensitivity to etoposide, facilitating the induction of pyroptosis (Fig. S1, *A*–*D*). Etoposide treatment in SH-SY5Y and A549 cells resulted in concentration-dependent cleavage of GSDME and caspase-3 (Fig. 1, *G* and *H*). Consistent with these observations, LDH release was observed (Fig. 1, *A* and *B*), along with characteristic pyroptotic morphology, indicated by cell swelling, large bubble formation on the plasma membrane (Fig. S1*E*), and increased percentages of PI^+^ and Annexin V^+^ cells (Fig. 1, *D*–*F*). These results confirmed etoposide-induced pyroptosis in SH-SY5Y and A549 cells.

Gene Expression Profiling Interactive Analysis (GEPIA) showed lower OGA expression in lung adenocarcinoma (LUAD), lung squamous cell carcinoma (LUSC), and glioblastoma multiforme (GBM) compared to standard samples, indicating increased O-GlcNAcylation in tumors (Fig. 1*L*). Low OGT mRNA expression was also associated with improved overall survival in LUSC patients (Fig. 1*C*). These findings suggest dysregulated O-GlcNAcylation in these tumors. Etoposide treatment inhibited elevated O-GlcNAcylation in tumors (Fig. 1, *I*–*K*), indicating that O-GlcNAcylation may correlate with etoposide-induced pyroptosis.

### O-GlcNAcylation levels negatively correlate with chemotherapy-induced pyroptosis

**Figure 2:**
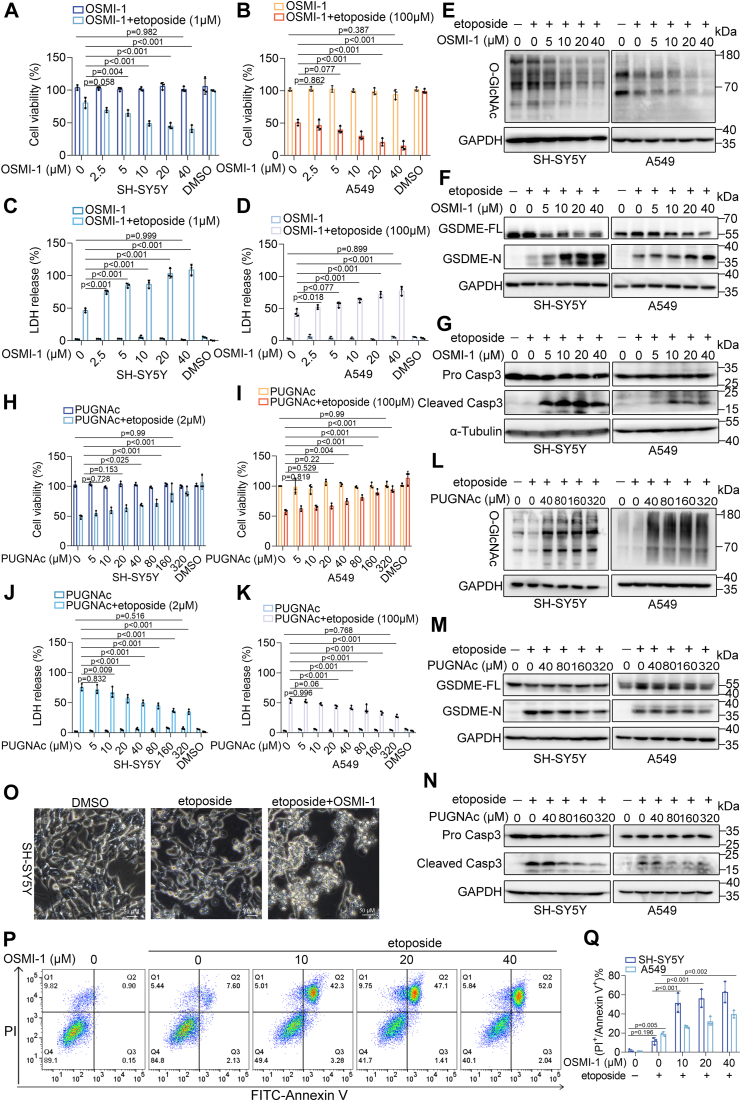
**O-GlcNAcylation regulates cell sensitivity to pyroptosis.***A* and *B*, cell viability of SH-SY5Y (*A*) and A549 cells (*B*) treated with different concentrations of OSMI-1, alone or in combination with etoposide. *C* and *D*, LDH release in SH-SY5Y (*C*) and A549 cells (*D*) treated with different concentrations of OSMI-1, alone or in combination with etoposide. *E*–*G*, Western blot analysis of O-GlcNAc (*E*), GSDME (*F*), and caspase-3 (*G*) in SH-SY5Y and A549 cells treated with etoposide or etoposide + OSMI-1. *H* and *I*, cell viability of SH-SY5Y (*H*) and A549 cells (*I*) treated with different concentrations of PUGNAc, alone or in combination with etoposide. *J*–*K*, LDH release in SH-SY5Y (*J*) and A549 cells (*K*) treated with different concentrations of PUGNAc, alone or in combination with etoposide. *L*–*N*, Western blot analysis of O-GlcNAc (*L*), GSDME (*M*), and caspase-3 (*N*) in SH-SY5Y and A549 cells treated with etoposide or etoposide + PUGNAc. *O*, representative microscopic images of SH-SY5Y cells treated with etoposide or etoposide + OSMI-1 for 12 h. Scale bar: 50 μm. *P*, flow cytometry analysis of Annexin V and PI staining in SH-SY5Y cells treated with etoposide or etoposide + OSMI-1 for 12 h shows the double-positive cell percentage. *Q*, quantification of the percentage of double-positive cells from panel (*P*). Data are presented as mean ± SD (n = 3), with significance determined by two-way ANOVA.

Cells were treated with various pharmacological inhibitors to investigate the role of O-GlcNAcylation in etoposide-induced pyroptosis. OSMI-1 treatment inhibited O-GlcNAcylation, while PUGNAc treatment increased it dose-dependently (Fig. S2, *A*–*F*). OSMI-1 treatment alone did not affect pyroptosis but significantly increased the sensitivity of cells to etoposide-induced pyroptosis (Fig. 2, *A*–*D*). The combination of etoposide and OSMI-1 significantly increased pyroptosis in a concentration-dependent manner, as indicated by increased LDH release (Figs. 2, *C* and *D*, S3, *C* and *D*), GSDME and caspase-3 cleavage (Fig. 2, *E*–*G*), pyroptotic cell morphology (Figs. 2*O*, S3*I*), and higher percentages of Annexin V^+^ and PI^+^ cells (Figs. 2*P*, S3*J*). The improved effect of this combination treatment was more significant at lower etoposide concentrations, indicating increased sensitivity to tumor therapy (Figs. 2, *A* and *B*, S3, *A* and *B*). In contrast, combination treatment with etoposide and PUGNAc decreased the sensitivity of cells to pyroptosis. PUGNAc pretreatment maintained cell viability after etoposide exposure (Figs. 2, *H* and *I*, S3, *E* and *F*), reduced LDH release (Figs. 2, *J* and *K*, S3, *G* and *H*), and decreased the percentage of Annexin V^+^ and PI^+^ cells (Fig. S3*J*). Phase-contrast images also supported these observations (Fig. S4*I*), and GSDME and caspase-3 cleavage were notably reduced following etoposide + PUGNAc treatment (Fig. 2, *L*–*N*).

To further validate these results, stable cell clones with *OGA* knockdown (sh-OGA), *OGT* knockdown (sh-OGT), *OGA* overexpression (ex-OGA), and *OGT* overexpression (ex-OGT) were established. Transfection efficiency was confirmed *via* western blotting (Fig. S2, *G*–*Q*). Compared to parental cells, sh-OGT, and ex-OGA cells showed reduced O-GlcNAcylation levels, increasing sensitivity to pyroptosis, as evidenced by increased LDH release, GSDME cleavage, and higher percentages of Annexin V^+^ and PI^+^ cells (Figs. S4, S5). In contrast, sh-OGA and ex-OGT cells showed opposite effects (Figs. S4, S5). These findings suggest that O-GlcNAcylation plays a vital role in regulating the sensitivity of tumor cells to etoposide-induced pyroptosis.

### Decreased O-GlcNAcylation leads to pyroptosis-mediated tumor regression

**Figure 3:**
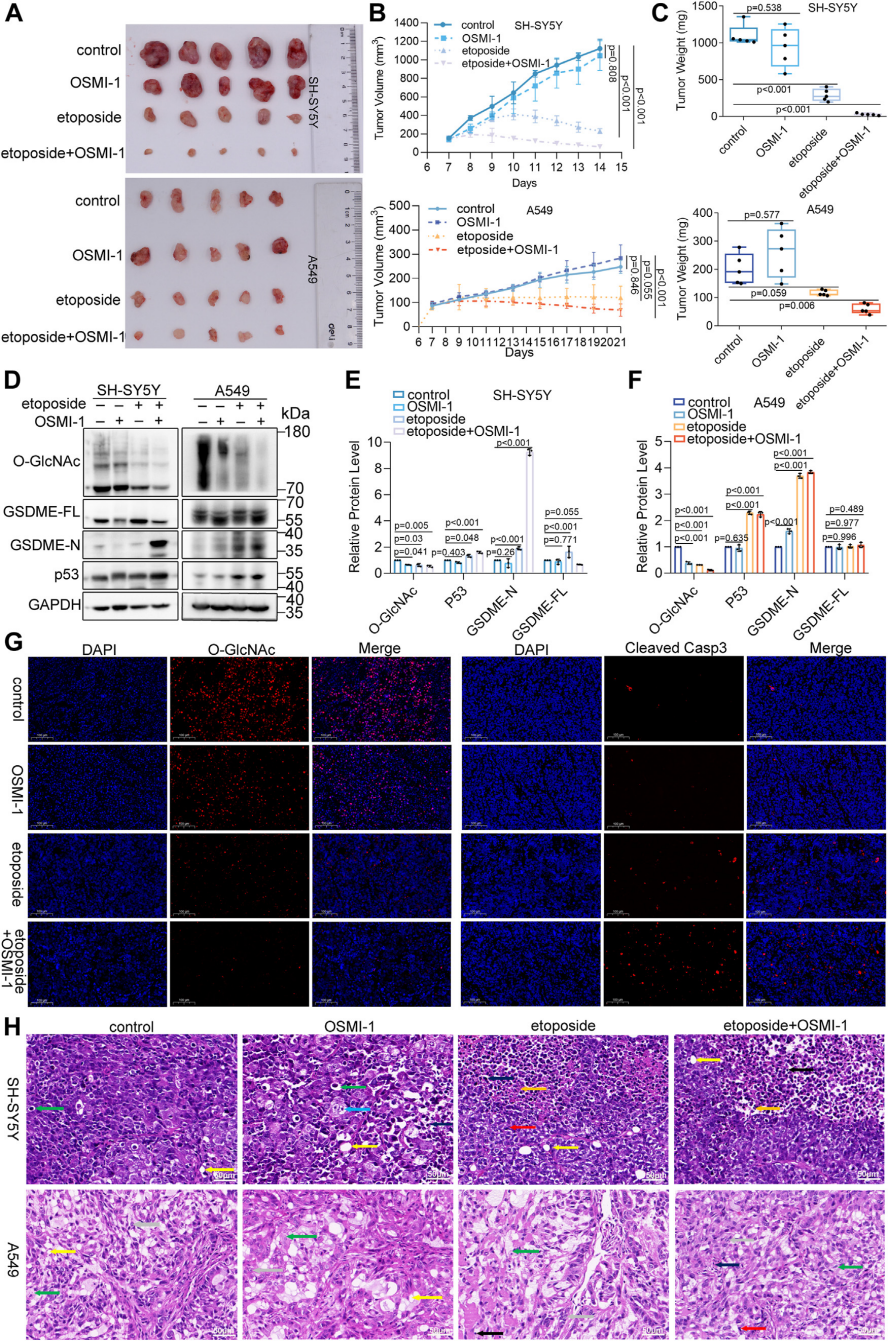
**Synergistic inhibition of human xenograft tumor growth by etoposide and OSMI-1 combination treatment.***A*, representative images of SH-SY5Y xenograft tumors (*upper panel*) and A549 xenograft tumors (*lower panel*) excised from nude mice. *B*, tumor growth in nude mice subcutaneously inoculated with SH-SY5Y and A549 cells. Mice were monitored for tumor growth for 7 days and then treated with DMSO, etoposide (20 mg/kg), OSMI-1 (10 mg/kg), or their combination by intraperitoneal injection once daily for 1 to 2 weeks. Tumor size was recorded daily, and growth curves were plotted. Data are presented as mean ± SD (n = 5). *C*, tumor weight statistics for SH-SY5Y xenografts (*upper panel*) and A549 xenografts (*lower panel*). Quantification is shown as mean ± SD (n = 5), with significance determined by one-way ANOVA. *D*, Western blot analysis of O-GlcNAc, GSDME, and p53 expression in xenograft tumors. *E* and *F*, relative expression levels of O-GlcNAc, GSDME, and p53 proteins in xenograft tumors. Data are presented as mean ± SD (n = 3), with significance determined by two-way ANOVA. *G*, immunofluorescence analysis of SH-SY5Y tumor sections treated with DMSO, etoposide, OSMI-1, or etoposide + OSMI-1, using antibodies against cleaved caspase-3 and O-GlcNAc. Scale bar: 100 μm. *H*, immunohistochemical analysis of pathological changes in xenograft tumors treated with DMSO, etoposide, OSMI-1, or etoposide + OSMI-1. *Green* arrows indicate tumor cell edema and degeneration; *yellow* arrows indicate round vacuoles; *blue* arrows indicate nuclear pyknosis; *red* arrows indicate pathological mitotic phases; *black* arrows indicate inflammatory cell infiltration; *orange* arrows indicate nuclear fragments; *gray* arrows indicate nucleocytoplasmic separation. Scale bar: 50 μm.

To evaluate the role of O-GlcNAcylation in etoposide-induced pyroptosis, xenograft mouse models were employed. Compared with controls, OSMI-1 treatment alone showed a mild inhibitory effect on tumor growth, whereas etoposide significantly reduced tumor size by 79.6% and 57.8%. The combination of etoposide and OSMI-1 therapy further enhanced the inhibition, reducing tumor size by 94.6% and 75.7% (Fig. 3, *A* and *B*). Furthermore, combination therapy significantly reduced tumor weight compared to both control and etoposide-treated groups (Fig. 3*C*). Immunohistochemical analysis indicated increased necrosis and inflammatory cell infiltration in tumors treated with the combination therapy, while etoposide or OSMI-1 alone resulted in minimal or no changes (Fig. 3*H*). O-GlcNAcylation levels were significantly reduced in tumors treated with either OSMI-1, etoposide, or the combination, with the combination therapy showing the most significant decrease. Moreover, GSDME activation was higher in the combination therapy than in etoposide alone (Fig. 3, *D*–*F*). Immunofluorescence assays further confirmed these findings, showing decreased O-GlcNAcylation and increased caspase-3 activation following combination treatment compared to individual treatments (Figs. 3*G*, S6*A*). These results suggest that combined etoposide and OSMI-1 treatment increases caspase-3–GSDME-mediated pyroptotic signaling, inhibiting tumor growth.

### P53 is critical for etoposide-induced tumor cell pyroptosis

**Figure 4:**
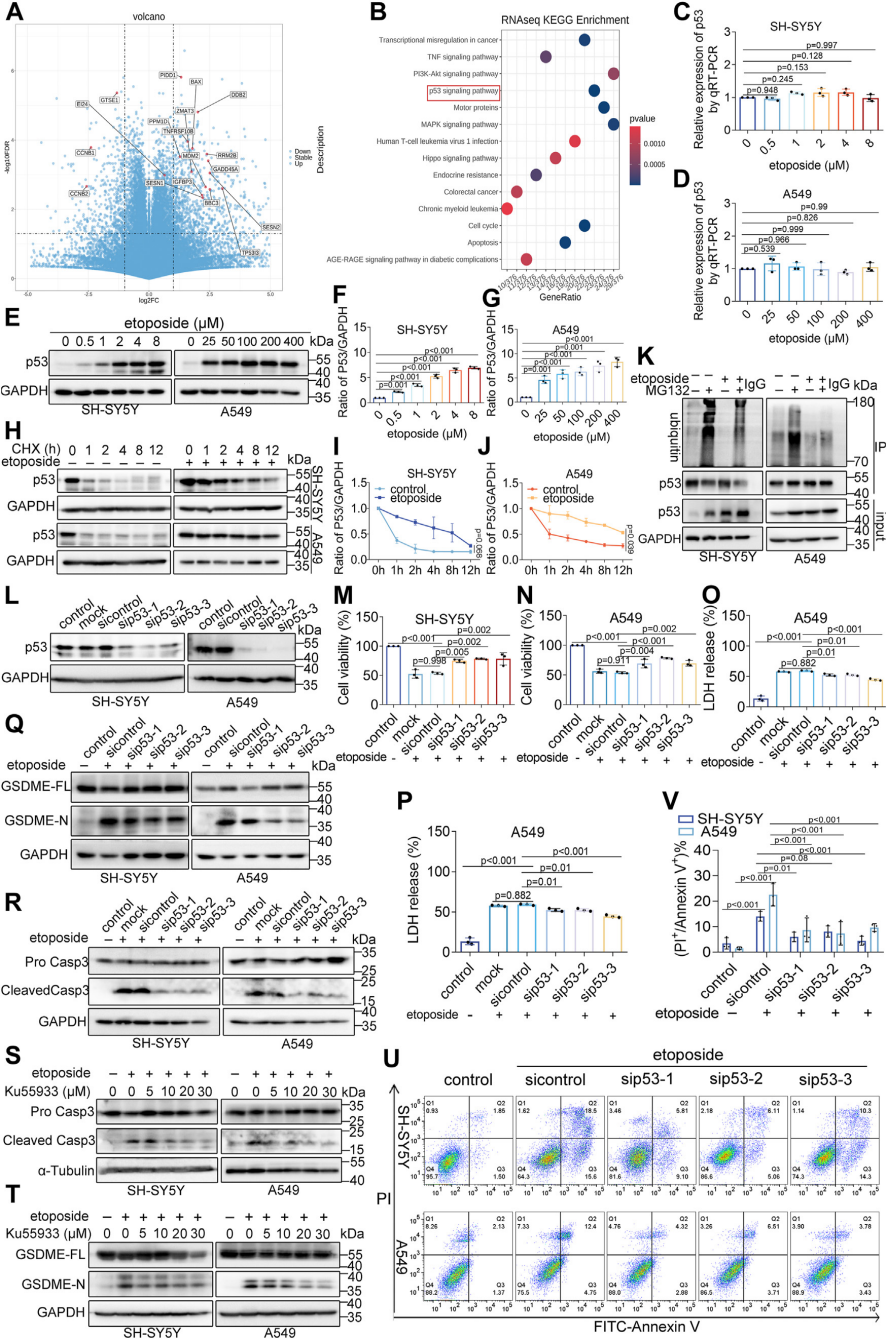
**P53 promotes etoposide-induced pyroptosis.***A*, Volcano plot of differentially expressed genes in etoposide-treated *versus* untreated SH-SY5Y cells. *B*, KEGG analysis of differentially expressed genes identified from panel (*A*). *C* and *D*, p53 mRNA expression in SH-SY5Y (*C*) and A549 cells (*D*) after treatment with different concentrations of etoposide. *E*, Western blot analysis of p53 protein levels after treatment with various concentrations of etoposide. *F* and *G*, relative expression levels of p53 protein. *H*, Western blot analysis of p53 stability after treatment with Cycloheximide (CHX, 100 μg/ml) for different durations in untreated cells (*left*) and etoposide-pretreated cells (*right*). *I* and *J*, quantification of p53 protein stability from panel (*H*). Data are presented as mean ± SD, using unpaired two-tailed *t* test, with n = 3 biological replicates. *K*, Western blot analysis of p53 ubiquitination in SH-SY5Y and A549 cells after treatment with 40 μM MG132 for 6 h. *L*, validation of p53 knockdown efficiency in cells. *M* and *N*, CCK8 assay measuring cell viability of SH-SY5Y (*M*) and A549 cells (*N*) after etoposide treatment in negative control (sicontrol), blank control (mock), and p53 interference (sip53) groups. *O* and *P*, percentage of LDH release from SH-SY5Y (*O*) and A549 cells (*P*) after etoposide treatment in negative control (sicontrol), blank control (mock), and p53 interference (sip53) groups. *Q*–*R*, Western blot analysis of GSDME (*Q*) and caspase-3 (*R*) in control (sicontrol) and p53 interference (sip53) groups after etoposide treatment. *S* and *T*, Western blot analysis of GSDME (*S*) and caspase-3 (*T*) in cells treated with etoposide alone and combined with Ku55933. *U*, flow cytometry analysis measures Annexin V^+^ and PI^+^ cell percentages in etoposide-treated control and p53 interference (sip53) groups. *V*, quantification of Annexin V^+^ and PI^+^ cells from panel (*U*). Data are presented as mean ± SD (n = 3), with significance determined by two-way ANOVA.

To further investigate the mechanism of etoposide-induced pyroptosis, transcriptomic analysis *via* RNA sequencing was performed on SH-SY5Y cells treated with or without etoposide. The volcano plot indicated 1079 differentially expressed genes, with 715 genes upregulated and 364 genes downregulated following etoposide treatment (Fig. 4*A*). Principal component analysis (PCA) showed a distinct separation between etoposide-treated and control cells (Fig. S7*G*). GO and KEGG analyses revealed that differentially expressed genes were enriched in the p53 signaling pathway (Figs. 4*B*, S7*A*). The p53 interaction network analysis identified 55 differentially expressed genes involved in p53-mediated pathways, including Fas, PIDD, DR-5, Bax, and Puma (Fig. S7*B*). RT-qPCR results were consistent with transcriptomic findings, showing no change in p53 mRNA levels during pyroptosis (Fig. 4, *C* and *D*). However, p53 protein levels increased concentration-dependent (Fig. 4, *E*–*G*). This discrepancy suggests a differential regulation of p53 protein synthesis and degradation. Western blot analysis indicated that etoposide treatment reduced p53 protein degradation and extended its half-life, whereas p53 was rapidly degraded under normal conditions (Fig. 4, *H*–*J*). Etoposide also reduced p53 ubiquitination, with MG132 treatment significantly increasing p53 levels and its ubiquitination (Fig. 4*K*), suggesting p53 involvement in etoposide-induced pyroptosis.

*P53* was silenced using siRNAs to investigate the role of p53 in pyroptosis (Fig. 4*L*). *p53* knockdown significantly increased cell viability following etoposide treatment (Fig. 4, *M* and *N*), along with decreased LDH release (Fig. 4, *O* and *P*) and reduced Annexin V^+^ and PI^+^ cell percentages (Fig. 4, *U* and *V*). Consistent with these findings, caspase-3 and GSDME cleavage were significantly reduced in p53-silenced cells (Fig. 4, *Q* and *R*). It is well established that ATM phosphorylation and activation lead to increased p53 expression upon DNA damage (35). ATM inhibition with ku55933 yielded similar results (Figs. 4, *S* and *T*, S7, *C*–*F*, *H*). In contrast, p53 overexpression promoted concentration-dependent pyroptosis, as evidenced by increased LDH release (Fig. S8, *C* and *D*), GSDME and caspase-3 cleavage (Fig. S8, *E*–*J*), and higher percentages of Annexin V^+^ and PI^+^ cells (Fig. S8, *K*–*M*), thereby increasing etoposide sensitivity (Fig. S8, *A* and *B*). These results indicate that p53 plays a crucial role in etoposide-induced pyroptosis.

### Caspase-3 activity is essential for p53-mediated pyroptosis

**Figure 5:**
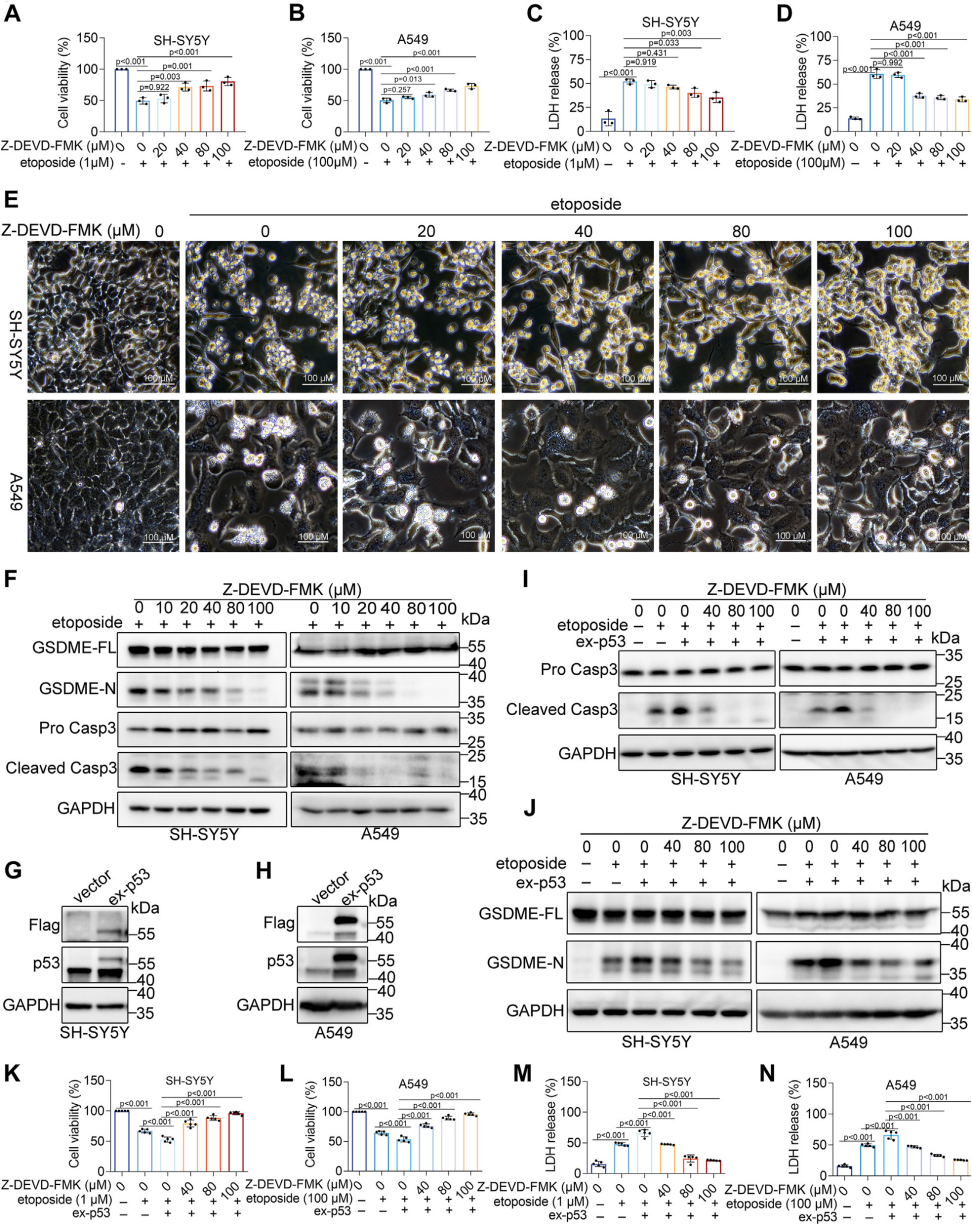
**P53-mediated pyroptosis is dependent on caspase-3 activity.***A* and *B*, CCK8 assay measuring the cell viability of SH-SY5Y (*A*)s and A549 cells (*B*) in etoposide and etoposide + Z-DEVD-FMK treatment groups. *C* and *D*, LDH release assay in SH-SY5Y (*C*) and A549 cells (*D*) after etoposide and etoposide + Z-DEVD-FMK treatment. *E*, representative microscopic images of SH-SY5Y and A549 cells treated with etoposide or etoposide + Z-DEVD-FMK for the indicated time points. Scale bar: 100 μm. *F*, Western blot analysis of caspase-3 and GSDME cleavage in cells treated with etoposide or etoposide + Z-DEVD-FMK. *G* and *H*, validation of p53 overexpression efficiency in cells. *I* and *J*, Western blot analysis of GSDME and caspase-3 in vector and p53-overexpressing (ex-p53) cells treated with etoposide, with or without Z-DEVD-FMK. *K* and *L*, cell viability of SH-SY5Y (*K*) and A549 cells (*L*) measured after etoposide treatment in vector and ex-p53 cells, with or without Z-DEVD-FMK. *M* and *N*, percentage of LDH release in culture supernatants from vector and ex-p53 cells after etoposide treatment, with or without Z-DEVD-FMK. Data were analyzed using one-way ANOVA.

Previous studies have shown that activated caspase-3 cleaves GSDME, initiating pyroptosis (18, 19). This study found that p53 regulates caspase-3 activity, requiring an investigation into whether caspase-3 is required for p53-mediated pyroptosis. To elucidate this role, the caspase-3 inhibitor Z-DEVD-FMK was employed. Pre-incubation with Z-DEVD-FMK not only abolished etoposide-induced caspase-3 and GSDME cleavage (Fig. 5*F*), but also reduced LDH release (Fig. 5, *C* and *D*) and cell death (Fig. 5, *A* and *B*). Morphological analysis further supported these findings (Fig. 5*E*). Moreover, p53 overexpressing cell lines were constructed (Fig. 5, *G* and *H*). p53-mediated increases in cell death and LDH release were significantly blocked by Z-DEVD-FMK pretreatment (Fig. 5, *K*–*N*). Similarly, p53-mediated caspase-3 and GSDME cleavage were reduced considerably following Z-DEVD-FMK pretreatment (Fig. 5, *I* and *J*). These results indicate that caspase-3 activity is required for etoposide-induced pyroptosis mediated by p53.

### P53 O-GlcNAcylation controls etoposide-induced pyroptotic sensitivity by regulating its ubiquitination

**Figure 6:**
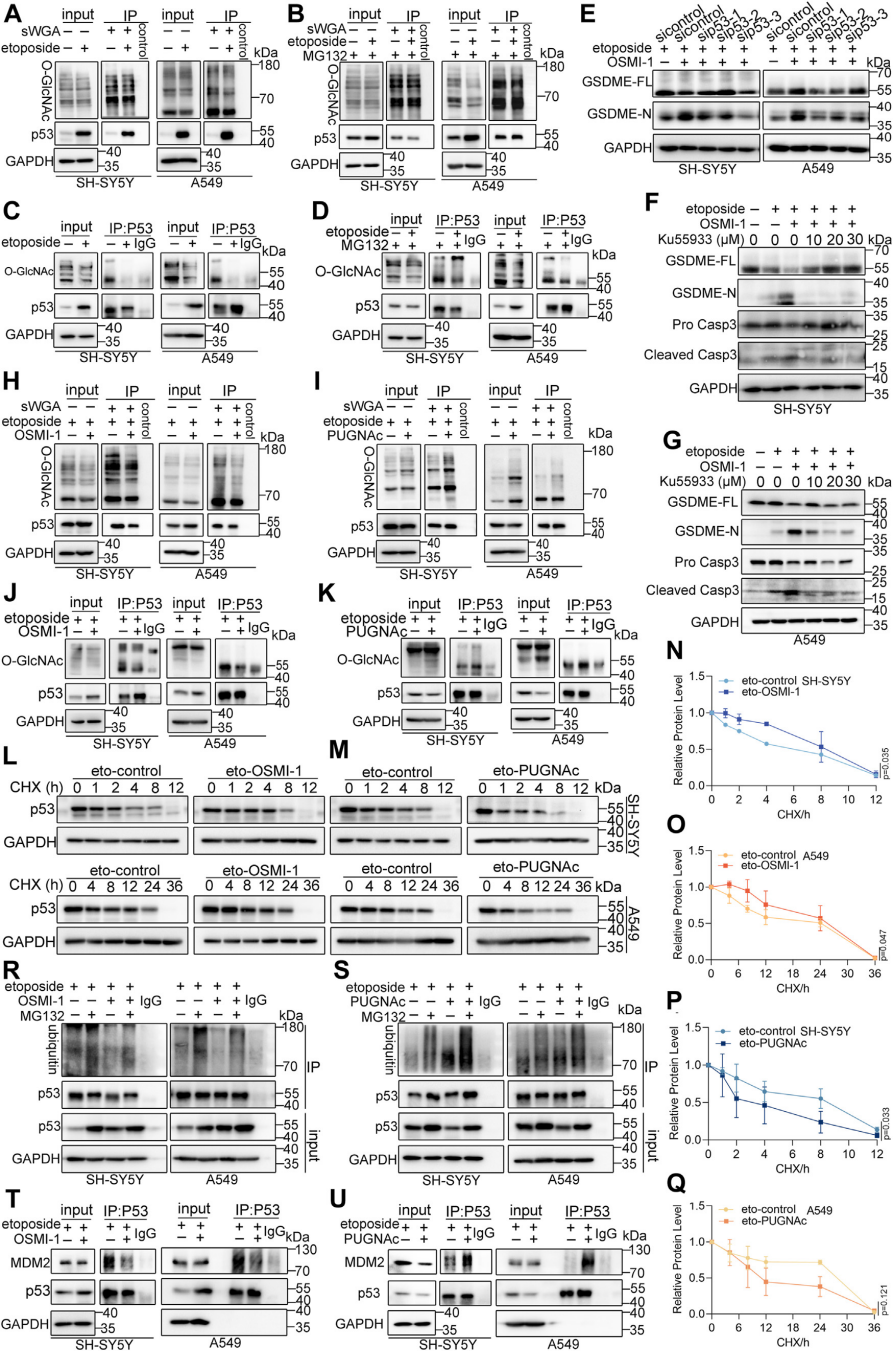
**O-GlcNAcylation of p53 was involved in etoposide-regulated tumor pyroptotic sensitivity.***A* and *B*, Western blot analysis of p53 and O-GlcNAcylation levels in SH-SY5Y and A549 cells captured by sWGA agarose or non-lectin conjugated agarose beads. The control refers to blank lysate + sWGA. *C* and *D*, Co-immunoprecipitation analysis detecting the O-GlcNAcylation of the p53 protein. *E*, SH-SY5Y and A549 cells were transfected with NC siRNA or p53 siRNA and treated with etoposide alone or combined with OSMI-1. GSDME cleavage was assessed by western blotting. *F* and *G*, Western blot detection of caspase-3 and GSDME cleavage in SH-SY5Y (*F*) and A549 (*G*) cells treated with etoposide, etoposide + OSMI-1, and etoposide + OSMI-1 + KU55933. *H* and *I*, Western blot analysis of p53 and O-GlcNAcylation levels in SH-SY5Y (*left*) and A549 (*right*) cells captured by sWGA agarose beads or non-lectin conjugated agarose beads under etoposide, etoposide + OSMI-1 (*H*), and etoposide + PUGNAc (*I*) treatments. *J* and *K*, immunoprecipitation analysis detecting O-GlcNAcylation of the p53 protein in SH-SY5Y (*left*) and A549 (*right*) cells treated with etoposide, etoposide + OSMI-1 (*J*), and etoposide + PUGNAc (*K*). *L* and *M*, Western blot analysis of p53 protein stability in etoposide and etoposide + OSMI-1 (*L*), and etoposide + PUGNAc (*M*) treatment groups. *N*–*Q*, quantification of panels (*L* and *M*), presented as mean ± SD, using unpaired two-tailed t-tests, with n = 3 biological replicates. *R* and *S*, Western blot analysis of p53 expression and ubiquitination in etoposide, etoposide + OSMI-1 (*R*), and etoposide + PUGNAc (*S*) treatment groups. *T* and *U*, SH-SY5Y and A549 cell extracts treated with etoposide, etoposide + OSMI-1 (*T*), and etoposide + PUGNAc (*U*) for the indicated times; total p53 and MDM2 levels were assessed by western blotting, and the interaction between p53 and MDM2 was analyzed by co-immunoprecipitation.

Previous studies have shown that O-GlcNAcylation affects protein stability (36, 37, 38). P53 stability increased significantly during pyroptosis, although p53 mRNA levels remained unchanged (Fig. 4, *C*–*J*). Yang *et al.* reported that O-GlcNAcylation at Ser149 affected p53 activity and stability in MCF-7 cells (39). sWGA pull-down assays were employed to confirm if p53 had O-GlcNAcylation to explore whether O-GlcNAcylation regulates p53 stability during pyroptosis (Fig. 6*A*). Immunoprecipitation analysis further revealed decreased O-GlcNAcylation of p53 during pyroptosis (Fig. 6*C*). MG132 was used to stabilize p53 protein to make the results more rigorous, and it was found that the O-GlcNAcylation of p53 was decreased by etoposide treatment (Fig. 6*D*). Elevated sWGA reactivity upon MG132 treatment suggested that p53 O-GlcNAcylation promotes its degradation (Fig. 6*B*). GEPIA database analysis indicated strong associations between p53 and O-GlcNAcylation-related genes (OGA and OGT) (Fig. S9, *Q*–*T*). To assess the role of p53 in O-GlcNAcylation-regulated pyroptosis, GSDME and caspase-3 cleavage were analyzed following p53 knockdown and inhibition. Inhibition of O-GlcNAcylation increased pyroptosis, but this effect was reduced in p53 knockdown cells (Fig. 6, *E*–*G*), suggesting that p53 plays a vital role in regulating pyroptosis *via* O-GlcNAcylation.

Further analysis revealed that OSMI-1 reduced p53 O-GlcNAcylation and its reactivity with sWGA, increasing its protein but not mRNA expression (Figs. 6, *H* and *J*; S9*A*). Consistent results were observed in ex-OGA cells (Fig. S9, *C*, *D* and *G*). In contrast, increased p53 O-GlcNAcylation decreased p53 protein expression, as seen in PUGNAc-treated and ex-OGT cells (Figs. 6, *I* and *K*; S9, *E*, *F* and *H*). Co-treatment with etoposide and OSMI-1 further increased p53 protein levels compared to etoposide alone *in vivo* (Fig. S6*B*). These results suggest that O-GlcNAcylation regulates p53 expression through translational or post-translational mechanisms. Furthermore, Cycloheximide (CHX) chase analysis revealed that p53 stability was impaired by PUGNAc treatment (Fig. 6, *M*, *P* and *Q*), whereas OSMI-1 increased p53 stability (Fig. 6, *L*, *N* and *O*). This effect was confirmed in ex-OGA and ex-OGT cells (Fig. S9, *I*–*N*).

MG132 treatment was employed to restore p53 protein levels to investigate the relationship between p53 degradation, O-GlcNAcylation, and the ubiquitin/proteasome pathway (Figs. 6, *R* and *S*, S9, *O* and *P*). The reduced O-GlcNAcylation induced by OSMI-1 inhibited p53 ubiquitination (Fig. 6*R*), whereas PUGNAc treatment significantly increased p53 ubiquitination (Fig. 6*S*). Similar results were observed in ex-OGA and ex-OGT cells (Fig. S9, *O* and *P*). It was hypothesized that OSMI-1 reduces p53 ubiquitination by affecting either the amount or the post-translational modification of MDM2, an E3 ubiquitin ligase, or by altering MDM2-p53 interactions. However, O-GlcNAcylation of MDM2 was not observed following OSMI-1 treatment (data not shown). Co-immunoprecipitation results revealed that OSMI-1 significantly reduced the interaction between MDM2 and p53, while the total MDM2 levels remained unchanged (Fig. 6*T*). Similar results were obtained in ex-OGA cells (Fig. S9*U*). In contrast, PUGNAc treatment increased the MDM2-p53 interaction (Fig. 6*U*), which was also observed in ex-OGT cell lines (Fig. S9*V*). These findings suggest that O-GlcNAcylation of p53 facilitates its ubiquitination-dependent degradation *via* MDM2.

### De-O-GlcNAcylation on S96 and S149 stabilizes p53 by decreasing MDM2 interaction

**Figure 7:**
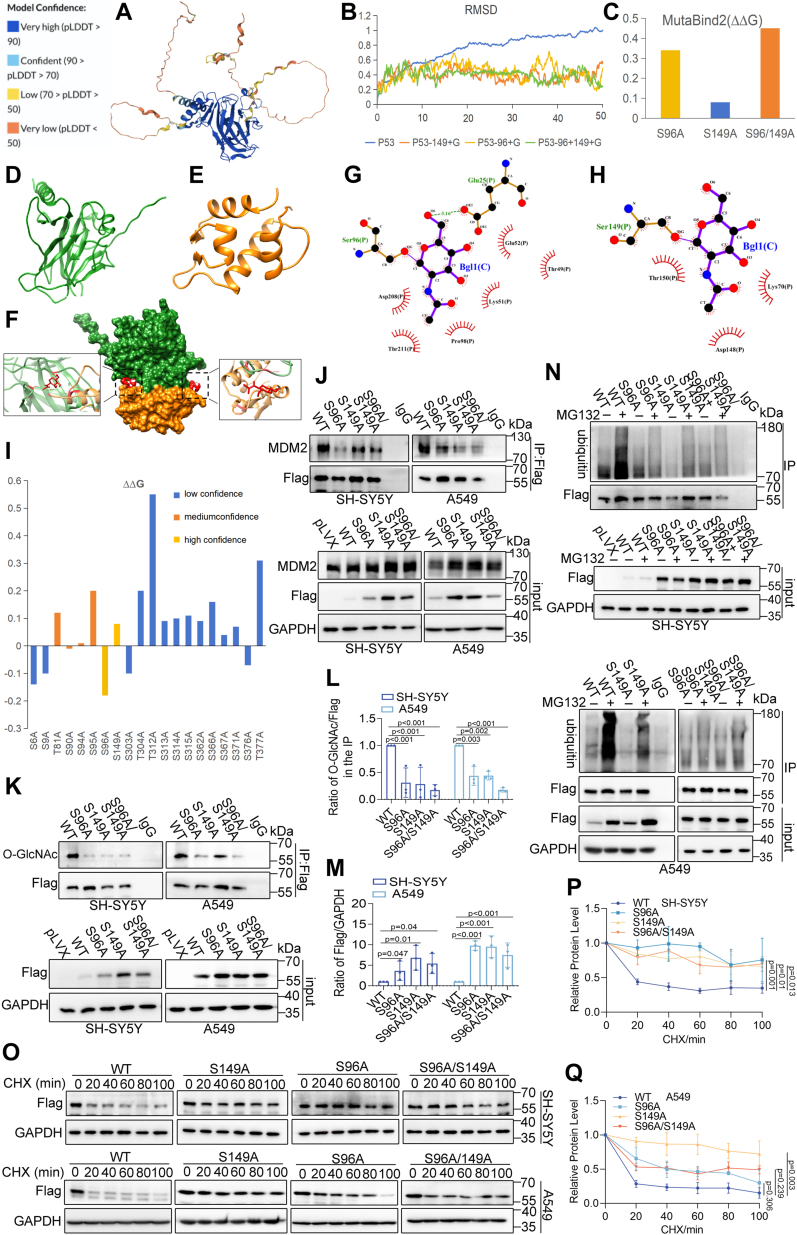
**P53 O-GlcNAcylation at S96 and S149 affects its stability.***A*, AlphaFold-generated per-residue confidence score (pLDDT) for p53, ranging from 0 to 100. *B*, RMSD of the p53 backbone over 50 ns of molecular dynamics (MD) simulation. *C*, MutaBind2-directed mutation analysis of potential O-GlcNAcylation sites on p53 to assess the structural stability of the p53-MDM2 complex. *D*, the truncated p53 structure predicted by AlphaFold shows high-confidence regions. *E*, structure of MDM2. *F*, combined p53 (*green*) and MDM2 (*orange*) conformation, with the O-GlcNAcylation sites at Ser96 and Ser149 shown in *red*. *G* and *H*, the binding pattern of the p53-MDM2 complex after O-GlcNAcylation at Ser96 and Ser149. *I*, PremPS-directed mutation analysis of potential glycosylation sites on p53 to assess structural stability. *J*, co-immunoprecipitation analysis of p53-MDM2 interaction in WT, S96A, S149A, and S96A/S149A cells. Control refers to the pLVX empty vector. *K*, co-immunoprecipitation analysis of O-GlcNAcylation in WT, S96A, S149A, and S96A/S149A cells (*upper panel*); total Flag levels were detected by western blotting (*lower panel*). *L* and *M*, quantifying of experimental data, presented as mean ± SD (n = 3), with significance determined by two-way ANOVA. *N*, Western blotting analysis of total Flag levels and ubiquitination of the Flag in WT, S96A, S149A, and S96A/S149A cells. *O*, Western blotting analysis of Flag stability in WT, S96A, S149A, and S96A/S149A cells. *P* and *Q*, quantification of data, presented as mean ± SD (n = 3), with significance determined by one-way ANOVA.

Potential O-GlcNAcylation sites of p53 were explored to investigate the role of p53 O-GlcNAcylation in regulating pyroptosis. Structural modeling was performed using AlphaFold to simulate mutant, non-glycosylated, and glycosylated variants of p53 (Fig. 7*A*). The NetOGlyc 4.0 server (http://www.cbs.dtu.dk/services/NetOGlyc/) identified 20 putative O-GlcNAcylation sites, and PremPS (40) was used to introduce targeted mutations. Among these, S96 and S149 were identified as high-reliability sites, demonstrating significant effects on p53 stability when mutated. S149 had previously been reported as an O-GlcNAcylation site (41), confirming the accuracy of these predictions (Fig. 7*I*). MD simulations were conducted using CHARMM-GUI (42). Analysis of root-mean-square deviation (RMSD) values indicated that O-GlcNAcylation at S96 and S149 increased p53 structural stability, while simultaneous O-GlcNAcylation at both sites did not significantly alter p53 structural stability (Fig. 7*B*). However, O-GlcNAcylation at S96 and S149 strengthened p53-MDM2 interactions. The MDM2 structure (residues 25–110) was obtained from PDB ID: 1RV1 (43), and the p53-MDM2 complex was generated using the Rosetta docking protocol (version 3.12) (44) (Fig. 7*F*). Among 100 generated poses during the docking process, one pose displayed favorable relative positioning, aligning the p53 domain near the MDM2 binding domain. MutaBind2 (41) was employed to introduce mutations, revealing that the loss of O-GlcNAcylation reduced the stability of the p53-MDM2 complex (Fig. 7*C*). LigPlot data indicated that O-GlcNAc at S96 and S149 increased contacts and hydrogen bonding at the p53-MDM2 binding surface, increasing the stability of the complex (Fig. 7, *G* and *H*). In summary, O-GlcNAcylation likely increases the interaction between p53 and MDM2 to promote p53 protein degradation.

**Figure 8:**
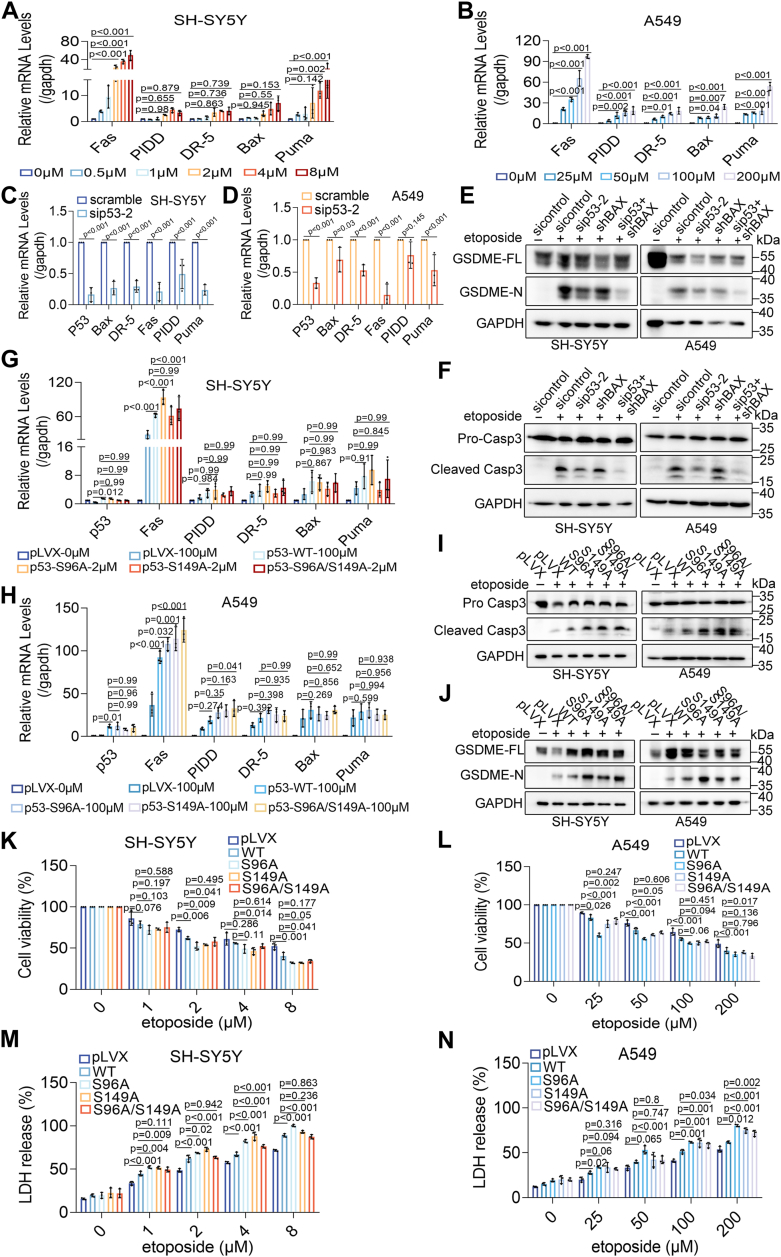
**The B-cell lymphoma 2 (Bcl-2) family and death receptor-related genes were involved in p53 O-GlcNAcylation-driven pyroptotic sensitivity.***A* and *B*, mRNA levels of Fas, PIDD, DR-5, Bax, and Puma in SH-SY5Y (*A*) and A549 (*B*) cells after treatment with different concentrations of etoposide. *C* and *D*, RT-qPCR analysis of Fas, DR-5, Bax, PIDD, Puma, and p53 expression in p53 interference (sip53) and control groups. *E* and *F*, Western blot analysis of GSDME cleavage (*E*) and caspase-3 cleavage (*F*) in control (sicontrol), p53 interference (sip53), BAX knockdown (shBAX), and p53 + BAX knockdown (sip53 + shBAX) groups after etoposide treatment. *G* and *H*, RT-qPCR analysis of p53, Fas, PIDD, DR-5, Bax, and Puma mRNA levels in WT, S96A, S149A, and S96A/S149A cells treated with etoposide. *I* and *J*, Western blot analysis of caspase-3 cleavage (*I*) and GSDME cleavage (*J*) in pLVX, WT, S96A, S149A, and S96A/S149A cells. *K* and *L*, cell viability in WT, S96A, S149A, and S96A/S149A cells treated with different concentrations of etoposide. *M* and *N*, LDH release in WT, S96A, S149A, and S96A/S149A cells after etoposide treatment. Data are presented as mean ± SD (n = 3), with significance determined by two-way ANOVA.

To determine the functional relevance of O-GlcNAcylation at these sites, S96A, S149A, and S96A/S149A mutants were generated by substituting alanine for serine at the respective sites on the Flag-p53 plasmid. Co-immunoprecipitation assays showed reduced O-GlcNAcylation in S96A, S149A, and S96A/S149A mutants compared to wild-type p53 (Fig. 7, *K* and *L*). Furthermore, p53 protein levels increased in S96A and S149A mutants without changes in mRNA levels (Fig. 8, *G* and *H*). Further analysis revealed that the p53-MDM2 interaction was weakened in these mutants, leading to decreased ubiquitination (Fig. 7, *J* and *N*) and increased p53 stability (Fig. 7, *O*–*Q*). These findings suggest that O-GlcNAcylation at S96 and S149 is vital for regulating p53 stability and interaction with MDM2 in SH-SY5Y and A549 cells. S96 may also be a putative O-GlcNAcylation site.

### The B-cell lymphoma 2 (Bcl-2) family/death receptor-related genes contribute to p53 O-GlcNAcylation-driven pyroptotic sensitivity

As a critical transcription factor, p53 exerts its biological effects through transcriptional regulation. To elucidate the underlying mechanism of p53 O-GlcNAcylation-regulated pyroptotic sensitivity, we assessed the expression of p53-interacting genes from the transcriptome. RT-qPCR analysis showed that the expression of p53, FAS, PIDD, DR-5, BAX, and PUMA was upregulated during pyroptosis (Fig. 8, *A* and *B*). In contrast, FAS, DR-5, PIDD, PUMA, and BAX expression decreased in p53 knockdown cells (Fig. 8, *C* and *D*) and increased in p53 overexpression cells (Fig. 8, *G* and *H*). BAX knockdown was performed to explore the role of BAX in p53-mediated pyroptosis (Fig. S9, *W* and *X*). Western blot analysis revealed that caspase-3 and GSDME cleavage was inhibited by BAX or p53 knockdown and further reduced by combined BAX and p53 knockdown (Fig. 8, *E* and *F*). The functional roles of p53 target genes in p53 O-GlcNAcylation-regulated pyroptotic sensitivity were further examined using various p53 mutants. Compared to wild-type p53, the S96A mutation led to a significant increase in FAS mRNA levels and a slight increase in PIDD, DR-5, and PUMA expression. In A549 cells, the S149A mutation increased FAS and PIDD expression, but this effect was not observed in SH-SY5Y cells, indicating cell-specific responses to the modification (Fig. 8, *G* and *H*). Further analyses showed that S96A and S149A mutations, compared to wild-type p53, decreased cell viability (Fig. 8, *K* and *L*) and increased LDH release (Fig. 8, *M* and *N*) under etoposide treatment. Immunoblotting confirmed that S96A and S149A mutants promoted caspase-3 and GSDME cleavage (Fig. 8, *I* and *J*). These findings suggest that de-O-GlcNAcylation of p53 enhances pyroptotic sensitivity through BCL2 family/death receptor-related genes.

## Discussion

O-GlcNAcylation, a post-translational modification, is frequently upregulated in tumors and disrupts cell signaling, transcriptional regulation, and cell cycle control, thereby contributing to tumorigenesis and metastasis (45, 46). This study observed increased O-GlcNAcylation in LUAD, LUSC, and GBM. Moreover, low OGT mRNA expression correlated with improved survival in LUSC patients. Previous studies have demonstrated that reducing O-GlcNAcylation in breast and colorectal cancer cells, among others, significantly inhibits tumor growth and migration (47, 48, 49, 50). Cellular stress, such as glucose deprivation, chemotherapeutic agents, and DNA damage, can significantly alter global O-GlcNAcylation levels, potentially regulating biological processes like apoptosis (51), autophagy (32), and ferroptosis (52). The findings from this study indicate that O-GlcNAcylation decreases during pyroptosis. Furthermore, inhibition of O-GlcNAcylation increased sensitivity to etoposide-induced pyroptosis by activating the caspase-3–GSDME signaling pathway, both *in vitro* and *in vivo*, while increased O-GlcNAcylation had the opposite effect. Thus, small-molecule inhibitors of O-GlcNAcylation may act as chemosensitizers, increasing the efficacy of chemotherapeutic drugs. Moreover, O-GlcNAcylation could serve as a biomarker for predicting the effectiveness of pyroptosis in refractory tumors, providing new strategies for cancer treatment.

At the molecular level, O-GlcNAcylation regulates protein activity, stability, subcellular localization, and protein-protein interactions, affecting cellular signaling pathways (36, 37, 38). Recent studies have demonstrated that O-GlcNAcylation of GSDMD reduces LPS-induced endothelial pyroptosis (53), whereas O-GlcNAcylation of NLRP3 enhances pyroptosis in human gingival fibroblasts (54). Transcriptomic analysis revealed enrichment of the p53 signaling pathway in the context of etoposide-induced pyroptosis. Despite unchanged p53 transcription levels, p53 protein levels increased, indicating the significance of post-translational modifications in regulating p53 stability. Results indicate that etoposide-induced pyroptosis is p53-dependent. Inhibition or knockdown of p53 reduced pyroptosis, whereas p53 overexpression increased pyroptotic activity. P53 function is closely regulated by its post-translational modifications (55). Post-translational modifications of p53 alter its chemical structure and interactions, increasing the diversity and complexity of p53-mediated cellular functions. Reduced O-GlcNAcylation of p53 decreased its interaction with MDM2, resulting in lower ubiquitination and increased protein stability. In contrast, increased O-GlcNAcylation of p53 promoted its destabilization.

This study identified that the S96A and S149A mutants reduced O-GlcNAcylation of p53, weakened its interaction with MDM2, and increased p53 stability. So S96 could be a putative O-GlcNAcylation site. However, the double mutation (S96A/S149A) had no synergistic effect, suggesting potential cross-talk with other modifications (39, 56, 57). The S96A and S149A mutants also upregulated the transcription of *Fas*, *DR-5*, *Puma*, and *PIDD*, which activate the death receptor signaling system, leading to the formation of the death-inducing signaling complex (DISC) and subsequent activation of the caspase cascade, ultimately resulting in caspase-3 activation (58). Moreover, activation of *Bax* and *Puma* triggers cytochrome C release from mitochondria, inducing apoptotic body formation and further activating caspase-3, which cleaves GSDME, initiating pyroptosis (18, 19). Interestingly, increased O-GlcNAcylation in lung cancer promotes p53 ubiquitination and degradation (59), while reduced O-GlcNAcylation stabilizes p53 and increases the efficacy of doxorubicin in hepatocellular carcinoma (31). In contrast, increased O-GlcNAcylation stabilizes p53 in ovarian cancer, facilitating its nuclear translocation (60). Yang *et al.* found that O-GlcNAcylation of p53 at Ser149 in MCF-7 cells can increase p53 stability (39). These variations may result from the high mutation rate of p53 (∼50%) in human tumors, leading to functional heterogeneity across cancer types (61). Even in patients with wild-type p53, a significant fraction shows a dysfunctional p53 pathway due to various factors (62). Different stress-inducing agents significantly differ in residue modification levels (63), as different stress signals can trigger distinct modification cascades (64, 65, 66). There is extensive cross-talk among p53 modifications (39, 56, 57), where some residues in p53 can be modified by different types, causing competition between modifying enzymes for the same site. For instance, acetylation of p53 C-terminal residues can block MDM2-mediated ubiquitination (67, 68), while phosphorylation at specific residues (Ser15, Thr18, Ser20) inhibits p53-MDM2 binding, preventing degradation (69, 70, 71). Phosphorylation of Thr155, induced by CSN, facilitates p53 ubiquitination *via* the ubiquitin-proteasome pathway (72, 73). New mass spectrometry methods for O-GlcNAc and metal ion affinity techniques for phosphorylation analysis have revealed extensive dynamic cross-talk between O-GlcNAcylation and phosphorylation (74, 75, 76). While individual modifications can fine-tune p53 function, combinations of modifications may modulate p53 activity in a promoter-specific manner, allowing diverse functional outcomes. Alterations in single-site modifications may produce tissue-, cell-type-, or stimulus-specific changes in p53 function. Therefore, the specific phenotypic changes resulting from post-translational modifications require careful investigation in mouse models. P53 modification is highly dynamic and context-dependent, serving as a central hub in coordinating responses to various cellular stresses.

**Figure 9:**
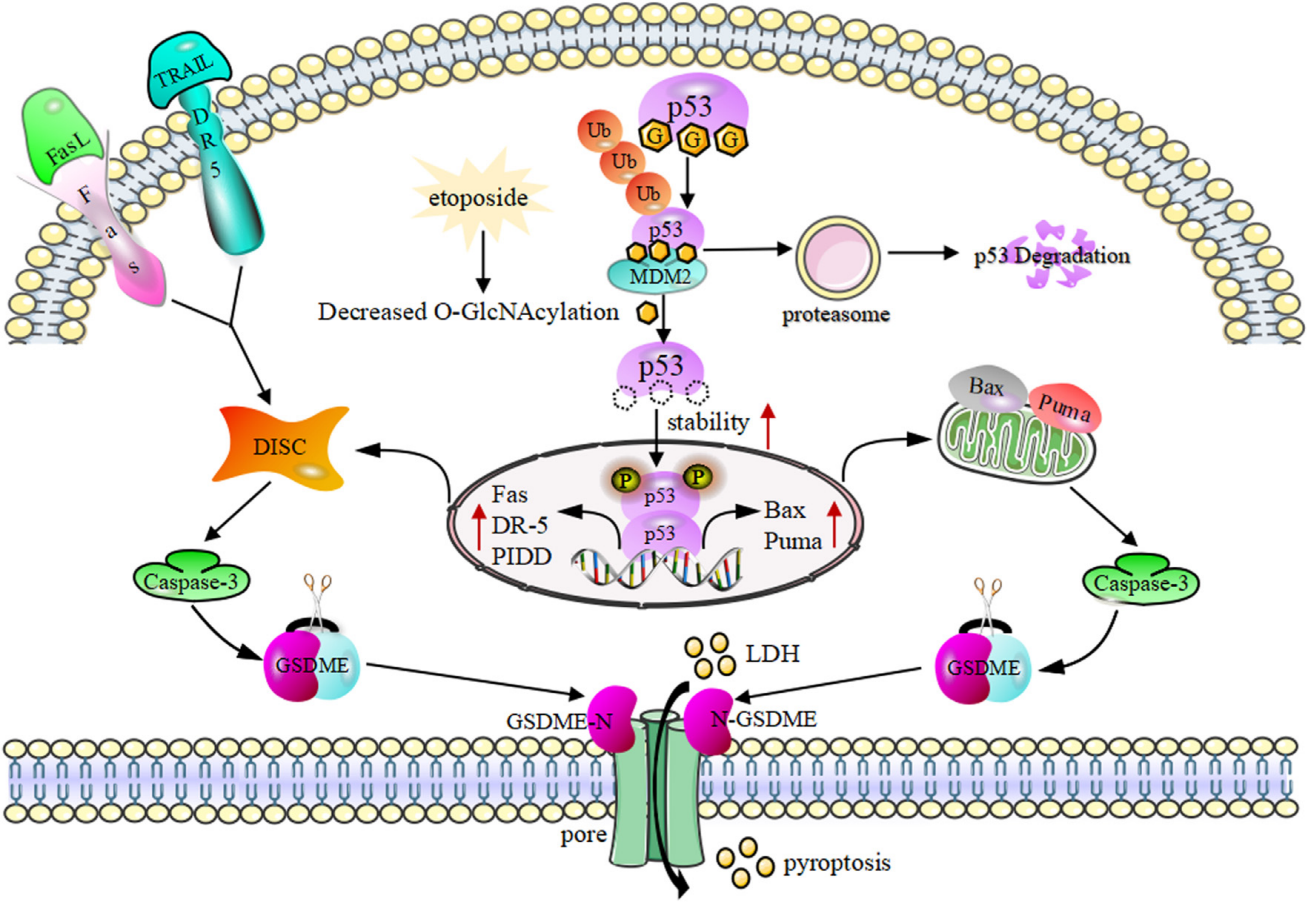
**Mechanistic diagram of dynamic O-GlcNAcylation regulating pyroptosis**.

In conclusion, this study elucidates a novel molecular mechanism highlighting the critical role of O-GlcNAcylation in modulating pyroptotic sensitivity. De-O-GlcNAcylation of p53 increases protein stability, leading to the transcriptional upregulation of the Bcl-2 family/death receptor-related genes. This cascade activates caspase-3-mediated cleavage of GSDME, thereby promoting pyroptosis (Fig. 9). Furthermore, identifying a putative O-GlcNAcylation site at S96 in p53 offers a potential therapeutic target for treating pyroptosis-related diseases.

## Experimental procedures

### Antibodies

The following antibodies were used in these studies. GSDME (Abcam, 215191); caspase-3 (Abcam, 184787); O-GlcNAc (Invitrogen, MA1-072); GAPDH (Proteintech, 60004-1-Ig); Goat anti-mouse IgG (Proteintech, SA00001–1); Goat anti-rabbit IgG (Proteintech, SA00001–2); α-Tubulin (Abcam, ab176560); P53 (Proteintech, 10442-1-AP); Rabbit anti-mouse IgG (Light Chain Specific) (Cell Signaling Technology, 58802S); Mouse anti-rabbit IgG (Conformation Specific) (Cell Signaling Technology, 5127S), Ubiquitin mouse mAb (Cell Signaling Technology, 3936S); OGA (Proteintech, 14711-1-AP); OGT (Proteintech, 11576-2-AP); MDM2 (Abcam, ab259265); DYKDDDDK tag antibody (Cell Signaling Technology, 2368S).

### Beads

The following beads were used in these immunoprecipitation analyses: Anti-DYKDDDDK Magnetic Agarose (Thermo Scientific, A36798); Succinylated WGA Agarose (Vector, AL-1023S); Dynabeads Protein A (Thermo Scientific, 10002D).

### Chemicals

Reagents were purchased as followings: Cell counting kit-8 (#C0038) from Beyotime Biotechnology; CytoTox 96 Non-Radio cytotoxicity assay kit (#G1780) from Promega; Annexin V-FITC/PI Apoptosis Detection Kit (#556547) from BD Biosciences; etoposide (#E1383), OSMI-1 (#SML1621), PUGNAc (#A7229) form Sigma; MG132 (#S2619), Cycloheximide (#S7418), Z-DEVD-FMK (#S7312), Ku55933 (#S7418) form Selleck; Lipofectamine RNAiMAX Reagent (#13778), Lipofectamine 2000 (#11668–019) form Invitrogen; Ceturegel Matrix LDEV-Free (#40183ES08) form Ye Sen; 4% paraformaldehyde (#BL539A) form Biosharp; Protease and Phosphatase Inhibitor Cocktail (#p002) form New Cell & Molecular Biotech; Puromycin (#A610593) from BBI Life Sciences Corporation.

### Cell culture

The human embryonic kidney cell line 293T, a human neuroblastoma cell line SH-SY5Y, human breast cancer cell lines HCC1937, T-47D, MDA-MB-231, MDA-MB-468, human hepatocellular carcinoma cell line HepG2, and lung cancer cell line A549 were purchased from Type Culture Collection of the Chinese Academy of Sciences. All cell lines were authenticated using a short tandem repeat analysis, and *mycoplasma* contamination testing was negative. All cells were cultured in DMEM (BasalMedia #L110KJ) or RPMI-1640 (BasalMedia #L210KJ) added with 10% fetal bovine serum (ExCell #FSP500) and 1% penicillin/streptomycin (New Cell & Molecular Biotech #C100C5). The cultures were maintained in a 5% CO2/95% air-humidified atmosphere at 37 °C.

### Plasmids and transfection

Packaging plasmid (psPAX2), envelope plasmid (pMD2.G), and lentivirus vector plasmid (pLKO.1/pLVX-puro) were all stored in our laboratory, and no mutations were found by sequencing. Then, the human OGT, OGA, and p53 genes were ligated into the pLVX-puro vector. The short hairpin RNAs (shRNA) targeting BAX, OGT, and OGA were cloned into the pLKO.1 vector; a scrambled shRNA was used as control. shRNA sequences are shown in Table S1. Flag-tagged p53 (S96A), Flag-tagged p53 (S149A), and Flag-tagged p53 (S96A/S149A) were constructed by site-directed mutagenesis using Vazyme's rapid mutation kit (#C214). All plasmids were verified by DNA sequencing. Lentiviral plasmids were transfected into HEK293T together with pMD2.G and psPAX2 according to the instructions of Lipofectamine 2000. After 48 h, the cell supernatant was collected and filtered to obtain a virus solution, which was further infected with SH-SY5Y and A549 cells. Puromycin was added 48 h after infection to obtain stable transfected cell lines.

### RNA interference

Gene Pharma synthesized all siRNAs used in this experiment, and the specific siRNA sequences are shown in Table S2. In brief, SH-SY5Y and A549 cells were seeded in 6-well plates at 2 × 10^5^ cells/well and prepared for transfection the next day when the cell density reached 70%. SiRNAs were transformed using Lipofectamine RNAiMAX according to the manufacturer's protocol. The transfected cells were cultured in an antibiotic-free medium for 24 h and then replaced with a fresh medium. After 48 h, the cells were collected to test the effect of siRNA interference or for subsequent experiments.

### Cell viability assays

Cells in the logarithmic phase were seeded in 96-well plates at a density of 1 × 10^4^ cells/well. The next day, different drugs were added for the indicated time. At the same time, a blank control group (medium only, drugs, no cells) and a negative control group (medium and cells, DMSO treatment) were set up. Five replicates were set for each treatment. Subsequently, 90 μl fresh medium and 10 μl CCK-8 solution were added to cells and incubated for 0.5 to 1 h at 37 °C. The absorbance value was measured at 450 nm using a microplate reader (Thermo Scientific). Cell viability (%) = (OD_drug treatment_ − OD_blank control_)/(OD_negative control_ − OD_blank control_) × 100%.

### LDH release assay

Cells grown in the logarithmic phase were seeded in 96-well plates at a density of 1 × 10^5^ cells/ml at 100 μl per well. When the cell density reached 50 to 60%, different drugs were added and treated for a specific time. At the same time, a negative control was set: no cells were used to exclude the medium background; LDH maximum release group: the same number of cells were seeded and treated with 10 μl 10 × Lysis Solution. The cell supernatant of the treatment, negative control, and LDH maximum release groups were collected. The release of LDH was measured according to the CytoTox 96 Non-Radio cytotoxicity assay kit instructions. Absorbance was measured at 490 nm using a microplate reader (Thermo Scientific). Each sample was tested in triplicates to obtain the average.

LDH release (%) = (OD_sample_ − OD_negative control_) / (OD_LDH maximum release group_ − OD_negative control_) × 100%

### Microscopy images

After the cells were treated with DMSO, etoposide, etoposide + OSMI-1, and etoposide + PUGNAc for the indicated time, they were placed under an Olympus inverted microscope and photographed for observation. Cells were first found at low magnification and then photographed and recorded under a 20x objective for 3 to 5 fields per treatment group.

### Western blotting

After drug treatment for the indicated times, cell precipitates were collected by centrifugation. A volume of RIPA lysis buffer (Beyotime Biotechnology, #P0013B) with a cocktail was added to the cell precipitate for lysis and sonication. After centrifugation at 12000*g* and 4 °C for 10 min, the protein concentration of the supernatant was measured by a BCA protein detection kit (Tian Gen #PA115). The samples were denatured in a loading buffer (New Cell & Molecular Biotech #WB2001). The samples were separated on sodium dodecyl sulfate-polyacrylamide gel electrophoresis (SDS-PAGE) and transferred to a PVDF membrane (Millipore). After being blocked for 1 h by 5% nonfat milk at room temperature, the membrane was incubated with primary antibodies overnight at 4 °C, followed by HRP-conjugated secondary antibodies for 1 h at room temperature. Then, the NcmECL Ultra (NCM Biotech, #P10200) was uniformly dropped onto the PVDF membrane and reacted in the dark for 5 min, and a BIORAD imager (ChemiDoc, Bio-Rad) was used for imaging. Protein quantification was performed using Image J software (National Institutes of Health).

### Flow cytometry

Cells in the logarithmic phase were seeded in 6-well plates at a density of 2.6 × 10^5^ cells/well. The next day, the corresponding drugs were added for the indicated time. After treatment, cells were washed twice with precooled PBS and resuspended in 1 × Annexin V binding buffer. A 100 μl cell suspension (1 × 10^5^ cells) was transferred to a 5 ml flow tube. After adding 5 μl Annexin V and 5 μl PI, cells were gently vortexed and incubated at room temperature for 15 min in the dark. 400 μl of 1 × Annexin V binding buffer was added to each flow tube. Data was obtained using flow cytometry (BD LSRfortessa) and analyzed by FlowJo software (Stanford University, USA).

### RNA-seq analysis

RNA-seq analysis was performed at Novogene Co., Ltd. In brief, total RNA was extracted from SH-SY5Y cells with or without etoposide treatment. After quality analysis, samples were submitted for cDNA library construction. Then, the prepared libraries were sequenced on an Illumina HiSeq 2000 platform. Clean reads were aligned to the reference sequence using Bowtie2 software, and then gene expression were calculated using RSEM software. Differential expression analysis was performed using the DESeq R software package. Genes with an adjusted *p*-value < 0.05 were considered differentially expressed. GO and KEGG enrichment analyses were performed on the predicted differential genes using the Cluster Profiler package in R language.

### Quantitative real-time PCR (RT-qPCR)

RNA was extracted according to the instructions of the RNAex Pro RNA extraction reagent (Accurate Biology, #AG21101), and the process was performed on ice to prevent degradation. 1 μg of total RNA was prepared for cDNA synthesis using Hifair III first Strand cDNA Synthesis SuperMix (Yeasen Biotechnology #HB210716). qPCR was performed using the CFX-96 real-time system (Bio-Rad) with qPCR SYBR Green Master Mix (Yeasen Biotechnology # HB220119). Primers used for amplification are shown in Table S3. Triplicate samples were used for each treatment and analyzed using a CFX-96 real-time system (Bio-Rad).

### Co-immunoprecipitation and lectin precipitation assay

For immunoprecipitation of endogenous p53 or exogenous Flag-p53 of WT/mutant for O-GlcNAcylation and ubiquitination examination, cells were lysed with Western and IP lysis buffer (Beyotime Biotechnology #P0013) supplemented with cocktail. Samples were sonicated three times for 10 s each on ice. After centrifugation, the supernatant was taken to determine protein concentration. 1 mg of protein was incubated with the indicated primary antibody overnight at 4 °C. Isotype control was also set: isotype control was added according to the amount of target antibody added. The next day, protein A magnetic beads or Anti-DYKDDDDK magnetic agarose were added to the protein-antibody complex and incubated for 6 h at 4 °C. The beads were resuspended in 1 × SDS loading buffer at 98 °C for 10 min and analyzed by western blotting.

Lectin precipitation assays were performed using the succinylated wheat germ agglutinin lectin (sWGA, which primarily binds to N-acetylglucosamine residues on proteins). The SH-SY5Y and A549 cells were lysed in a lysis buffer. The supernatant was denatured in glycoprotein denaturing buffer and digested with PNGase (P0704S; New England Biolabs) to remove N-linked glycoproteins. The 1 mg protein sample was incubated overnight with 100 μl sWGA in a vertical mixer at 4 °C. The next day, sWGA agarose beads were collected by centrifugation at 4 °C and washed three times with TBST (Tris Buffered Saline with Tween 20) for 10 min. Agarose beads were resuspended in 1 × SDS loading buffer and then boiled at 98 °C for 10 min for immunoblotting.

### Tumor xenograft assay

Balb/c-Nude female mice of 4 weeks old were fed in separate cages. The Ethical Review Committee approved the experiment for Animal Experiments of the College of Life Sciences, Sichuan University. SH-SY5Y and A549 cells in the logarithmic growth phase were harvested and resuspended in 50% PBS+ 50% Ceturegel Matrix LDEV-Free Matrigel. 100 μl cell suspension (5 × 10^6^) was injected subcutaneously into the right shoulder of nude mice. The tumor volume was calculated using the formula V = half × L × W^2^ (where L represents the length and W represents the width). When the tumor size reached 100 mm^3^, the nude mice were randomly divided into four groups according to 5 nude mice in each group. Drugs were prepared in a ratio of 5% DMSO + 40% PEG + 5% Tween-80 + 50% ddH_2_O. The mice were treated with etoposide (20 mg/kg), OSMI-1 (10 mg/kg), and etoposide + OSMI-1 (20 mg/kg + 10 mg/kg) by intraperitoneal injection. The tumor size was measured, and the body weight of each mouse was recorded. At the end of the experiment, the mice were sacrificed, and the tumor tissues were photographed and weighed. Part of the tumor tissues were fixed with 4% paraformaldehyde buffer, and immunohistochemical (IHC) and immunofluorescence (IF) staining were performed. Some tumor tissues were added to RIPA lysate for Western blot analysis.

### Immunofluorescence (IF) and Immunohistochemistry (IHC)

IF and IHC were performed according to the conventional protocols. Briefly, the tumor tissues were embedded in paraffin and made into slides. After deparaffinization, the slides were stained with hematoxylin-eosin and observed and photographed under a microscope. The primary antibodies used for IF were anti-p53 (Proteintech, 1:100), anti-O-GlcNAc (Invitrogen, 1:100), and anti-cleaved caspase3 (Abcam, 1:100). The slides were incubated with the secondary antibody of the corresponding species. Then, the nuclei were restained with DAPI. The slides were sealed and observed under the fluorescence microscope.

### Molecular dynamics (MD) simulations

The O-GlcNAc at Ser96 and Ser149 of the p53 fragment were modeled using the Glycan Reader and Modeler module (77). The impact of O-GlcNAcylation on the stability of the p53 fragment was investigated *via* molecular dynamics (MD) simulations performed with the GROMACS software (version 2021.2) (78). Three systems were considered: the mutated, unglycosylated, and O-GlcNAcylated fragments at Ser96 and Ser149. These systems utilized the TIP3P water model and the Amber ff99SB force field. 0.15 mol/L of sodium and chlorine ions were included to maintain electrical neutrality. The systems underwent minimization and equilibration following the default settings provided by the CHARMM-GUI web server (42). The steepest descent minimization procedure lowered the most potent force to 1000 kcal/(mol/nm). Subsequently, each system underwent 100 ps NVT and 100 ps NPT equilibrations at 300 K and 1 bar, with periodic boundary conditions consistently applied. The SHAKE algorithm was used to constrain bonds involving hydrogen atoms. MD trajectory data were analyzed using MDAnalysis, with backbone root mean square deviation (RMSD) calculated (79). The results were visualized using the xmgrace program. The interaction between the p53 fragment and the MDM2 was depicted using PyMol (80).

### Statistical analysis

All experiments were performed at least 3 times, and the experimental data were expressed as Mean ± SD. ANOVA was used for comparison among multiple groups by GraphPad Prism 5 software, and *p* < 0.05 and *p* < 0.01 represented statistically significant differences.

## Data availability

The gene expression dataset is available on the NCBI GEO repository (https://www.ncbi.nlm.nih.gov/geo/query/acc.cgi?acc=GSE266073), and the accession number is GSE266073.

## Supporting information

This article contains supporting information Figs. S1–S9 and Tables S1–S3.

## Conflict of interest

The authors declare the following financial interests/personal relationships which may be considered as potential competing interests.

Chuanfang Wu, Jinku Bao reports financial support was provided by the National Nature Science Foundation of China. Chuanfang Wu, Jinku Bao reports a relationship with National Natural Science Foundation of China that includes: funding grants. If there are other authors, they declare that they have no known competing financial interests or personal relationships that could have appeared to influence the work reported in this paper.
