# Supplementary material for: Dynamic O-GlcNAcylation coordinates etoposide-triggered tumor cell pyroptosis by regulating p53 stability
Source: J Biol Chem. 2024 Dec 10;301(1):108050. doi: 10.1016/j.jbc.2024.108050 (PMC11761933; doi:10.1016/j.jbc.2024.108050)
Supplement: Supplementary Information [file mmc1.pdf]

Supplementary Information

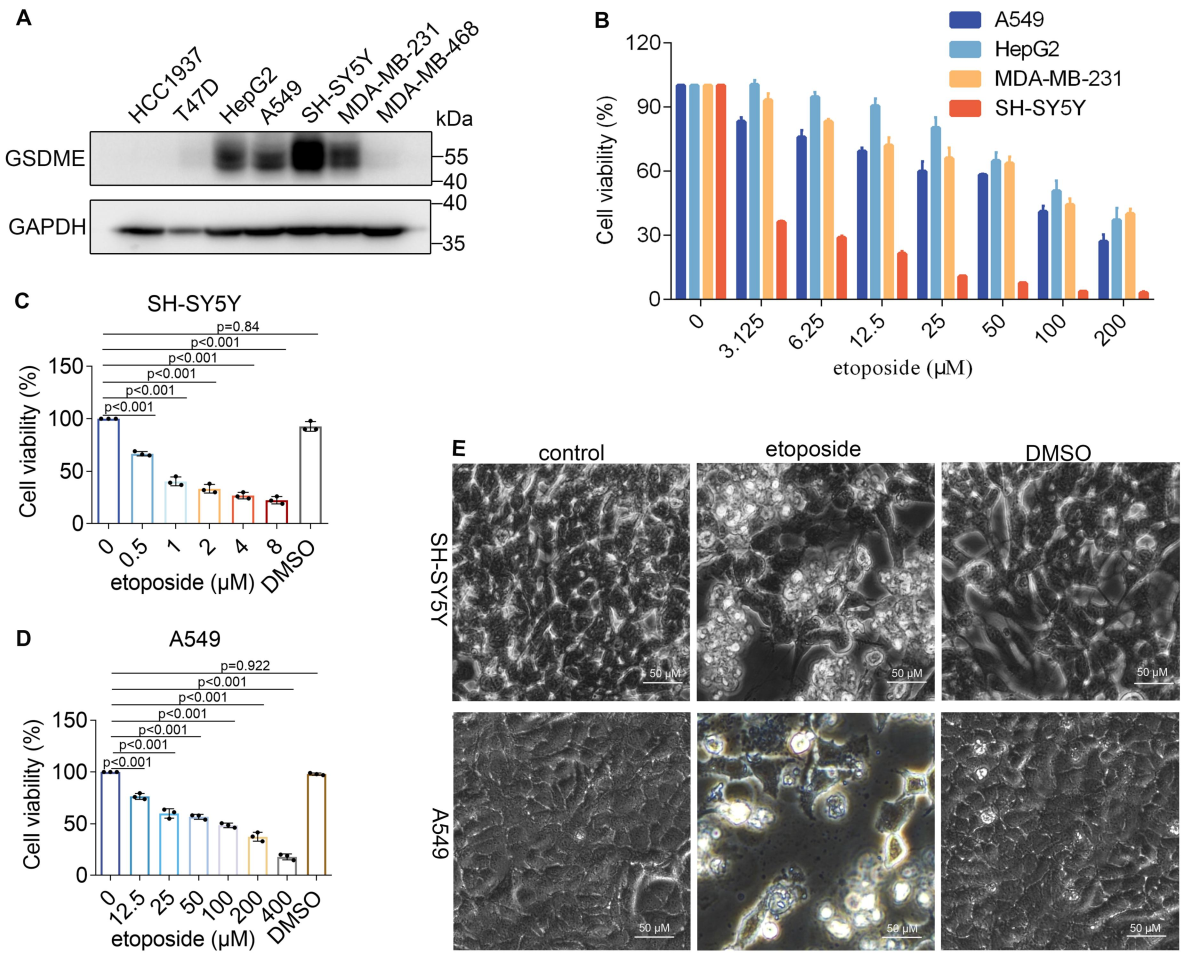

Figure S1. Etoposide induces pyroptosis of tumor cells. **A** Expresssion level of GSDME in various cell lines. **B** The cell viability of different cell lines was examined by etoposide treatment. **C** The cell viability of SH-SY5Y was detected after treatment with different concentrations of etoposide for 12 h. **D** The cell viability of A549 was detected after treatment with different concentrations of etoposide for 36 h. **E** After SH-SY5Y and A549 cells were treated with etoposide for indicated time, representative microscopic images were taken. Scale bar, 50 μm. Data represent mean ± SD (n = 3) with significance determined by two-way ANOVA.

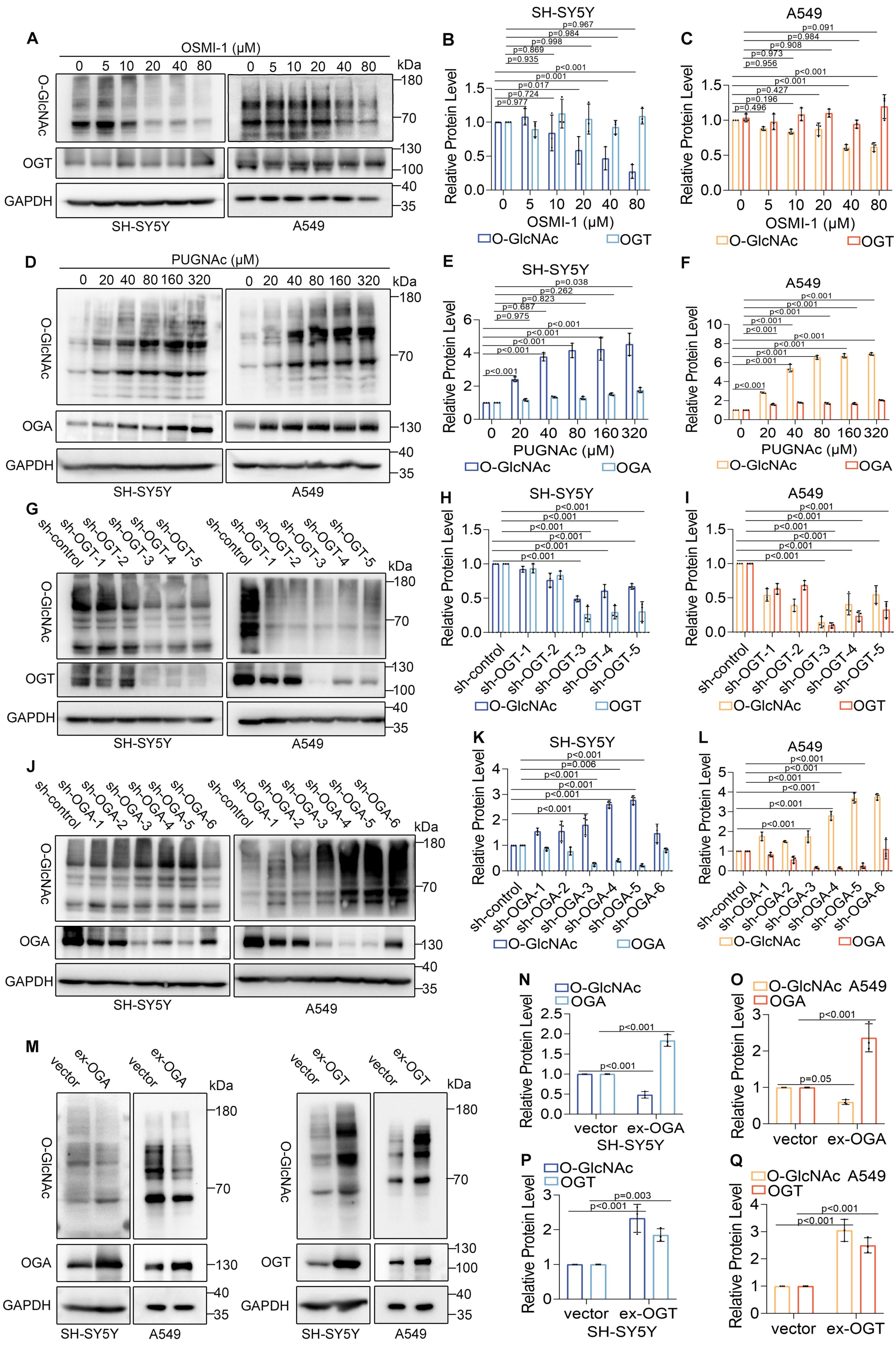

A
OSMI-1 (μM)
0 5 10 20 40 80 0 5 10 20 40 80
kDa
O-GlcNAc
OGT
GAPDH
180
70
130
100
40
35
SH-SY5Y
A549
B
SH-SY5Y
Relative Protein Level
OSMI-1 (μM)
O-GlcNAc
OGT
C
A549
Relative Protein Level
OSMI-1 (μM)
O-GlcNAc
OGT
D
PUGNAc (μM)
0 20 40 80 160 320 0 20 40 80 160 320
kDa
O-GlcNAc
OGA
GAPDH
SH-SY5Y
A549
E
SH-SY5Y
Relative Protein Level
PUGNAc (μM)
O-GlcNAc
OGA
F
A549
Relative Protein Level
PUGNAc (μM)
O-GlcNAc
OGA
G
sh-control
sh-OGT-1
sh-OGT-2
sh-OGT-3
sh-OGT-4
sh-OGT-5
kDa
O-GlcNAc
OGT
GAPDH
SH-SY5Y
A549
H
SH-SY5Y
Relative Protein Level
O-GlcNAc
OGT
I
A549
Relative Protein Level
O-GlcNAc
OGT
J
sh-control
sh-OGA-1
sh-OGA-2
sh-OGA-3
sh-OGA-4
sh-OGA-5
sh-OGA-6
kDa
O-GlcNAc
OGA
GAPDH
SH-SY5Y
A549
K
SH-SY5Y
Relative Protein Level
O-GlcNAc
OGA
L
A549
Relative Protein Level
O-GlcNAc
OGA
M
vector
ex-OGA
vector
ex-OGT
kDa
O-GlcNAc
OGA
OGT
GAPDH
SH-SY5Y
A549
N
O-GlcNAc
OGA
Relative Protein Level
p<0.001
p<0.001
vector
ex-OGA
SH-SY5Y
O
O-GlcNAc A549
OGA
Relative Protein Level
p<0.001
p=0.05
vector
ex-OGA
P
O-GlcNAc
OGT
Relative Protein Level
p=0.003
p<0.001
vector
ex-OGT
SH-SY5Y
Q
O-GlcNAc A549
OGT
Relative Protein Level
p<0.001
p<0.001
vector
ex-OGT

Figure S2. Efficiency verification of O-GlcNAcylation related inhibitors and plasmids. **A-C** O-GlcNAcylation expression in SH-SY5Y and A549 cells treated with different concentrations of OSMI-1. **D-F** O-GlcNAcylation expression in SH-SY5Y and A549 cells treated with different concentrations of PUGNAc. **G-I** SH-SY5Y and A549 cells were transfected with sh-control and sh-OGT plasmids, and the expression of OGT and O-GlcNAcylation were detected by western blotting. **J-L** SH-SY5Y and A549 cells were transfected with sh-control and sh-OGA plasmids, and the expression of OGA and O-GlcNAcylation were detected by western blotting. **M-Q** SH-SY5Y and A549 cells were transfected with vector, ex-OGT and ex-OGA plasmids, and the expression of OGT, OGA and O-GlcNAcylation were detected by western blotting. Data represent mean ± SD (n = 3) with significance determined by two-way ANOVA.

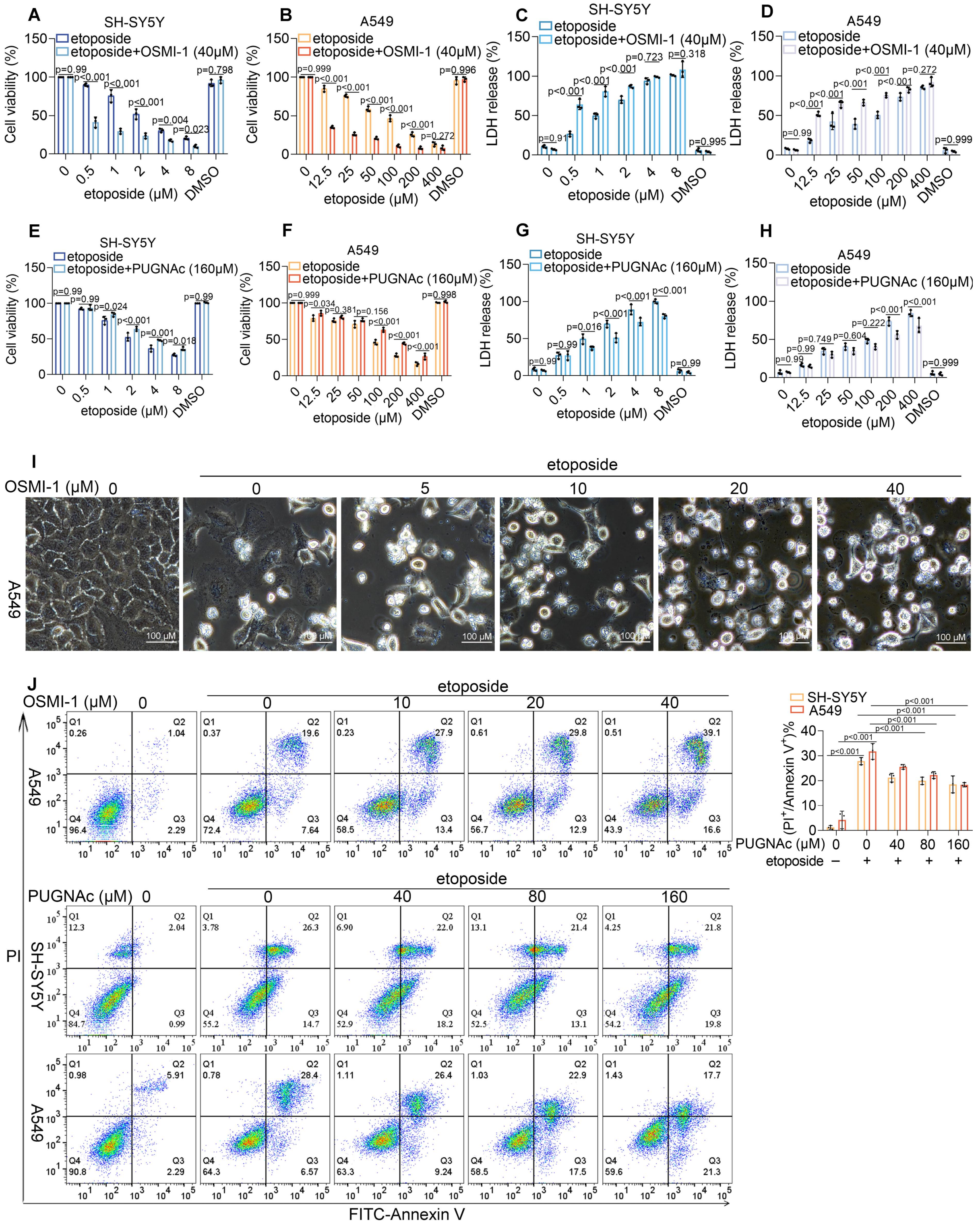

Figure S3. Inhibition of O-GlcNAcylation-related enzymes regulates the sensitivity of etoposide-induced pyroptosis. **A-B** The cell viability of SH-SY5Y (A) and A549 cells (B) were measured by different concentrations of etoposide alone or in combination with OSMI-1 (40 μM). **C-D** The LDH release of SH-SY5Y (C) and A549 cells (D) were measured by different concentrations of etoposide alone or in combination with OSMI-1 (40 μM). **E-F** The cell viability of SH-SY5Y (E) and A549 cells (F) were measured by different concentrations of etoposide alone or in combination with PUGNAc (160 μM). **G-H** The LDH release of SH-SY5Y (G) and A549 cells (H) were measured by different concentrations of

etoposide alone or in combination with PUGNAc (160 μM). **I** After A549 was treated with etoposide and etoposide + OSMI-1 for 36 h, representative microscopic images were taken. Scale bar, 100 μm. **J** After etoposide, etoposide + OSMI-1 and etoposide + PUGNAc treatment, cells were collected at the indicated time points and stained with Annexin V and PI. The percentages of double positive cells were detected by flow cytometry. Data represent mean ± SD (n = 3) with significance determined by two-way ANOVA.

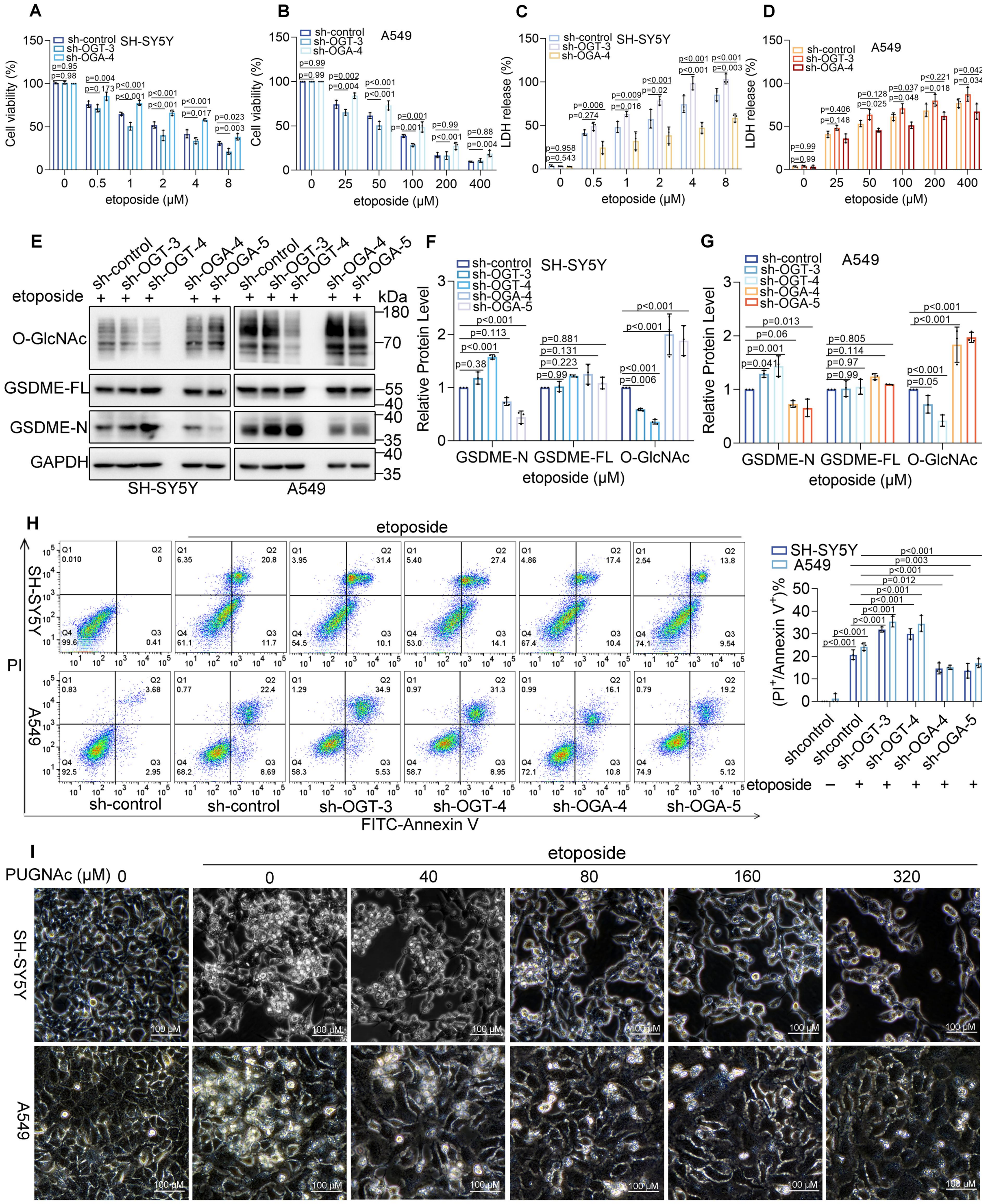

Figure S4. Interference of OGA and OGT regulates the sensitivity of etoposide-induced pyroptosis. **A-B** CCK-8 assay was used to detect cell viability in sh-control, sh-OGA and sh-OGT groups of SH-SY5Y (A) and A549 cells (B) after etoposide treatment. **C-D** LDH assay kit was used to detect LDH release in

sh-control, sh-OGA and sh-OGT groups of SH-SY5Y (C) and A549 cells (D) after etoposide treatment. **E-G** Western blotting was used to detect the O-GlcNAcylation and GSDME expression in sh-control and sh-OGT/sh-OGA cells treated with etoposide. **H** The percentages of AnnexinV and PI double-positive cells in the etoposide-treated sh-control and sh-OGT/sh-OGA cells were measured by flow cytometry. **I** After SH-SY5Y and A549 cells were treated with etoposide and etoposide + PUGNAc for indicated time, representative microscopic images were taken. Scale bar, 100 μm. Data represent mean ± SD (n = 3) with significance determined by two-way ANOVA.

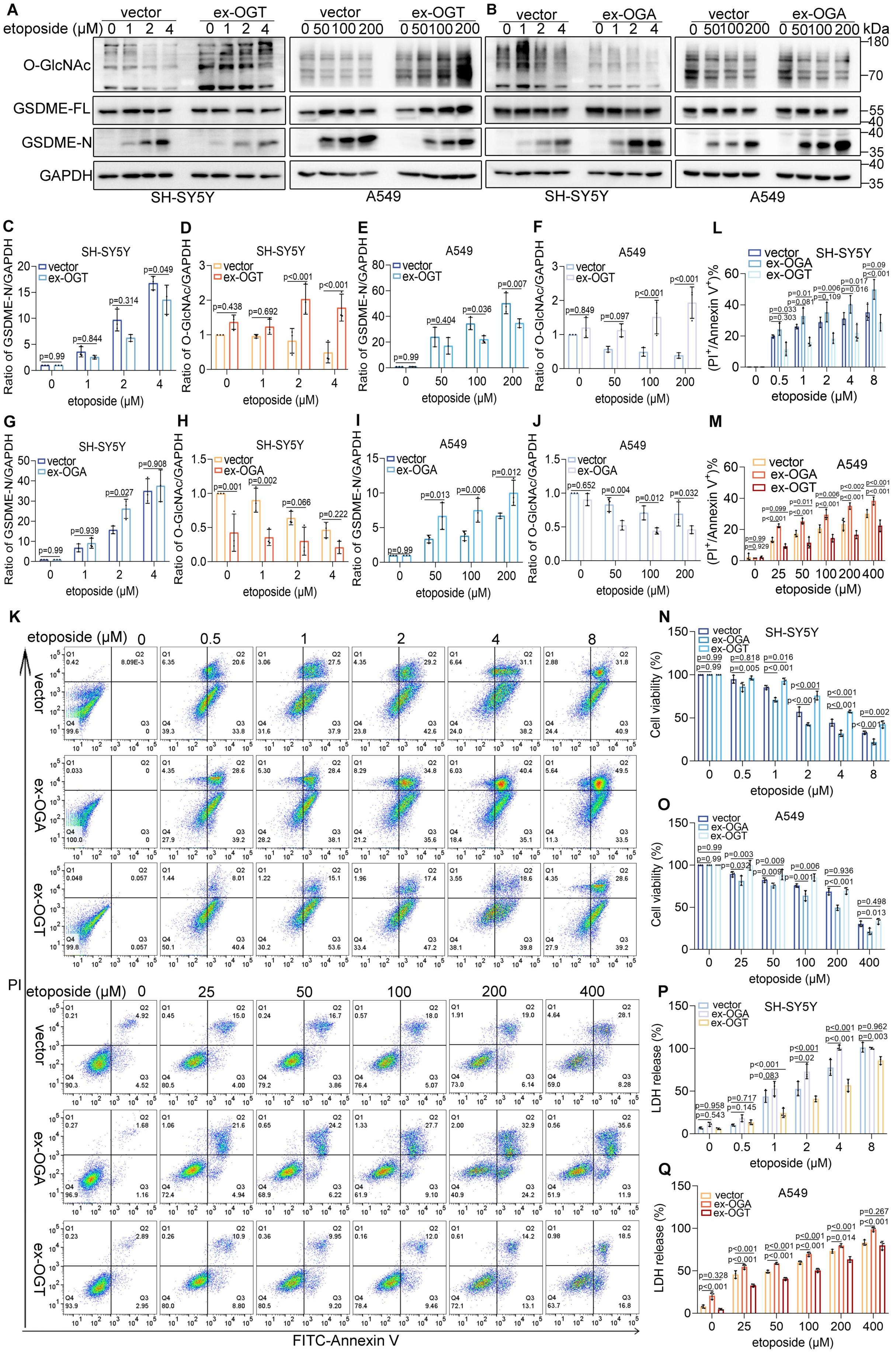

A
B
etoposide (μM)
vector
ex-OGT
ex-OGA
O-GlcNAc
GSDME-FL
GSDME-N
GAPDH
kDa
180
70
55
40
35
SH-SY5Y
A549
C
D
E
F
G
H
I
J
L
M
Ratio of GSDME-N/GAPDH
Ratio of O-GlcNAc/GAPDH
etoposide (μM)
(PI+/Annexin V+)%
K
PI
FITC-Annexin V
N
O
P
Q
Cell viability (%)
LDH release (%)

Figure S5. Overexpression of OGA and OGT regulate the sensitivity of etoposide-induced pyroptosis. **A-B** At the indicated time, immunoblottings of GSDME and O-GlcNAcylation were performed in vector, ex-OGA and ex-OGT cells treated with different concentrations of etoposide. **C-J** The relative expression of O-GlcNAcylation and GSDME in ex-OGA and ex-OGT cells compared with vector. **K** The percentages of AnnexinV and PI double-positive cells in the etoposide-treated vector, ex-OGA and ex-OGT cells were measured by flow cytometry. **L-M** Quantification of the percentages of double positive cells. **N-O** CCK-8 assay assessed the cell viability in vector, ex-OGA and ex-OGT groups of SH-SY5Y (N) and A549 cells (O) after etoposide treatment. **P-Q** LDH assay kit assessed LDH release in vector, ex-OGA and ex-OGT groups of SH-SY5Y (P) and A549 cells (Q) after etoposide treatment. Data represent mean ± SD (n = 3) with significance determined by two-way ANOVA.

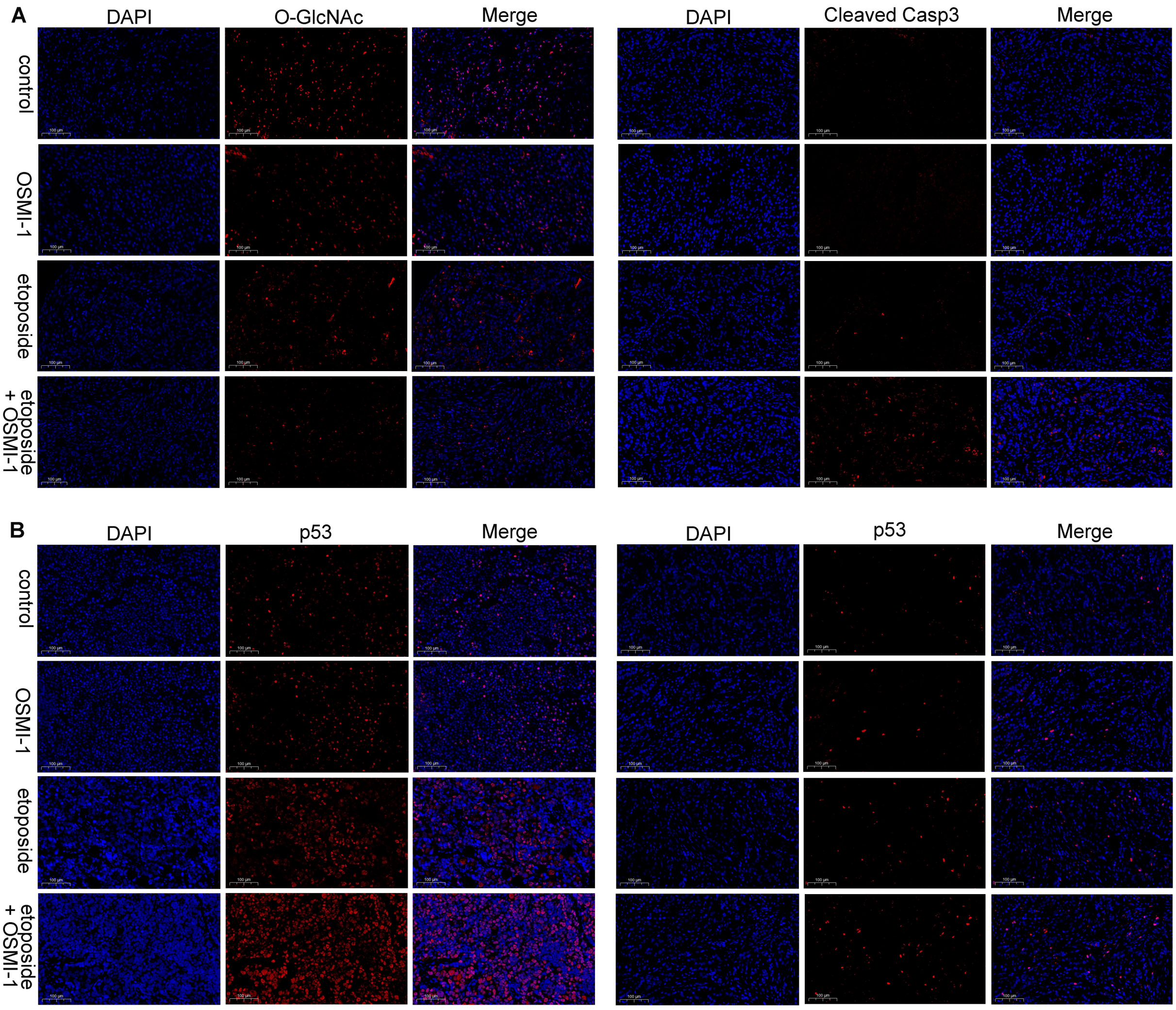

Figure S6. Immunofluorescence analysis of xenograft tumor associated protein. **A** Sections of the excised A549 tumors treated with DMSO, etoposide, OSMI-1, and etoposide + OSMI-1 were subjected to immunofluorescence analysis with antibodies against cleaved caspase-3 and O-GlcNAc. **B** Sections of the excised tumors treated with DMSO, etoposide, OSMI-1, and etoposide + OSMI-1 were subjected to immunofluorescence analysis with antibodies against p53. Scale bar: 100 μm.

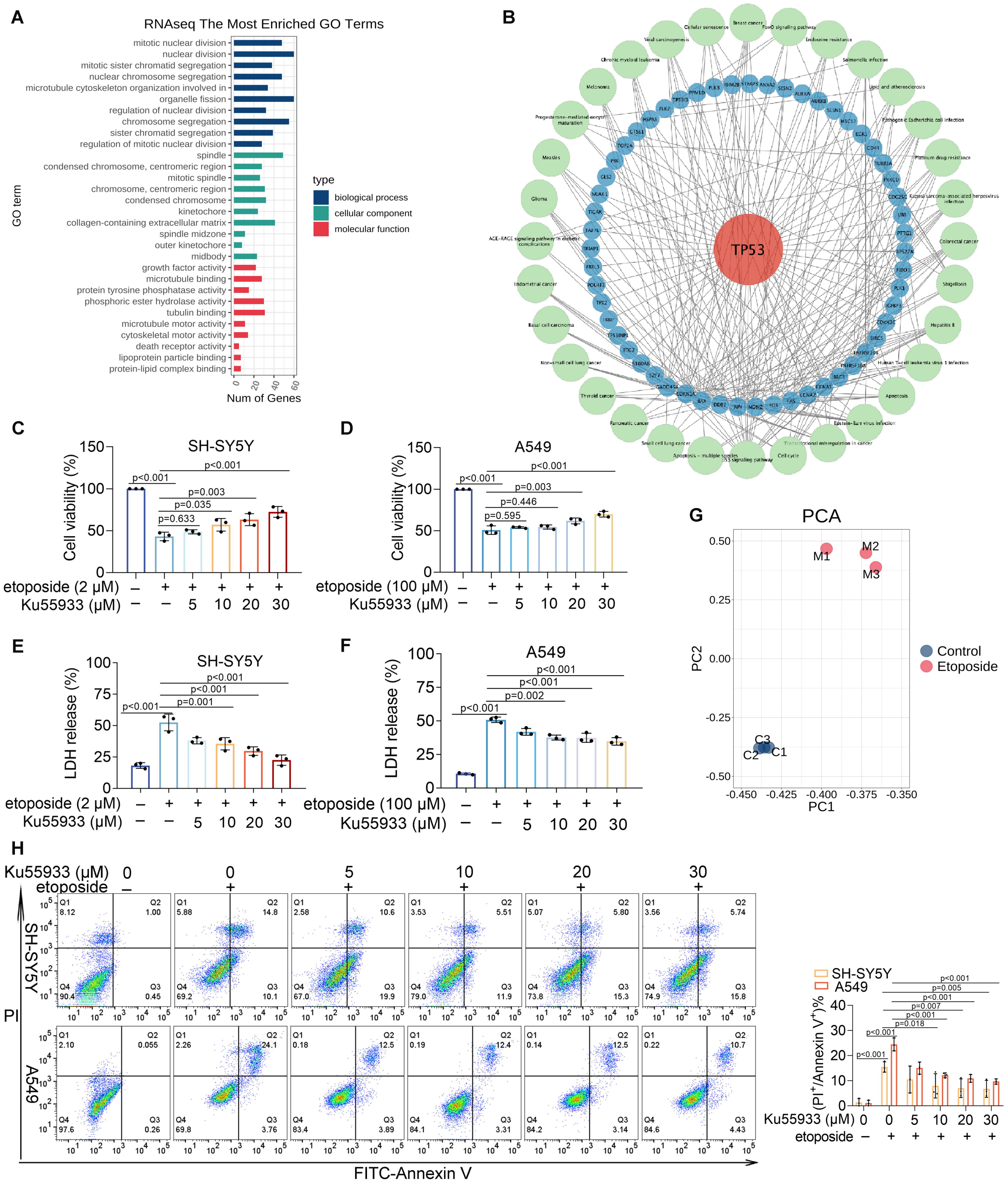

Figure S7. Inhibition of p53 activity inhibits etoposide-induced pyroptosis. **A** GO analysis of differentially expressed genes. **B** STRING protein-protein interaction analysis and functional enrichment analysis of p53 interacting with differentially expressed genes. **C-D** CCK8 was used to detect the cell viability of SH-SY5Y (C) and A549 cells (D) in etoposide treatment and etoposide + ku55933 treatment groups. **E-F** At the indicated time points, the percentage of LDH release in the culture supernatants from SH-SY5Y (E) and A549 cells (F) were measured after etoposide and etoposide + ku55933 treatment. Numbers of biological replicates are shown in their respective bars. Bars represent mean ± SD error. Comparisons across treatment groups were done with one-way ANOVA. **G** Principal component analysis (PCA) of identified genes. **H** The percentages of AnnexinV and PI double-positive cells in the etoposide-treated and

etoposide + ku55933-treated groups by flow cytometry. Data represent mean ± SD (n = 3) with significance determined by two-way ANOVA.

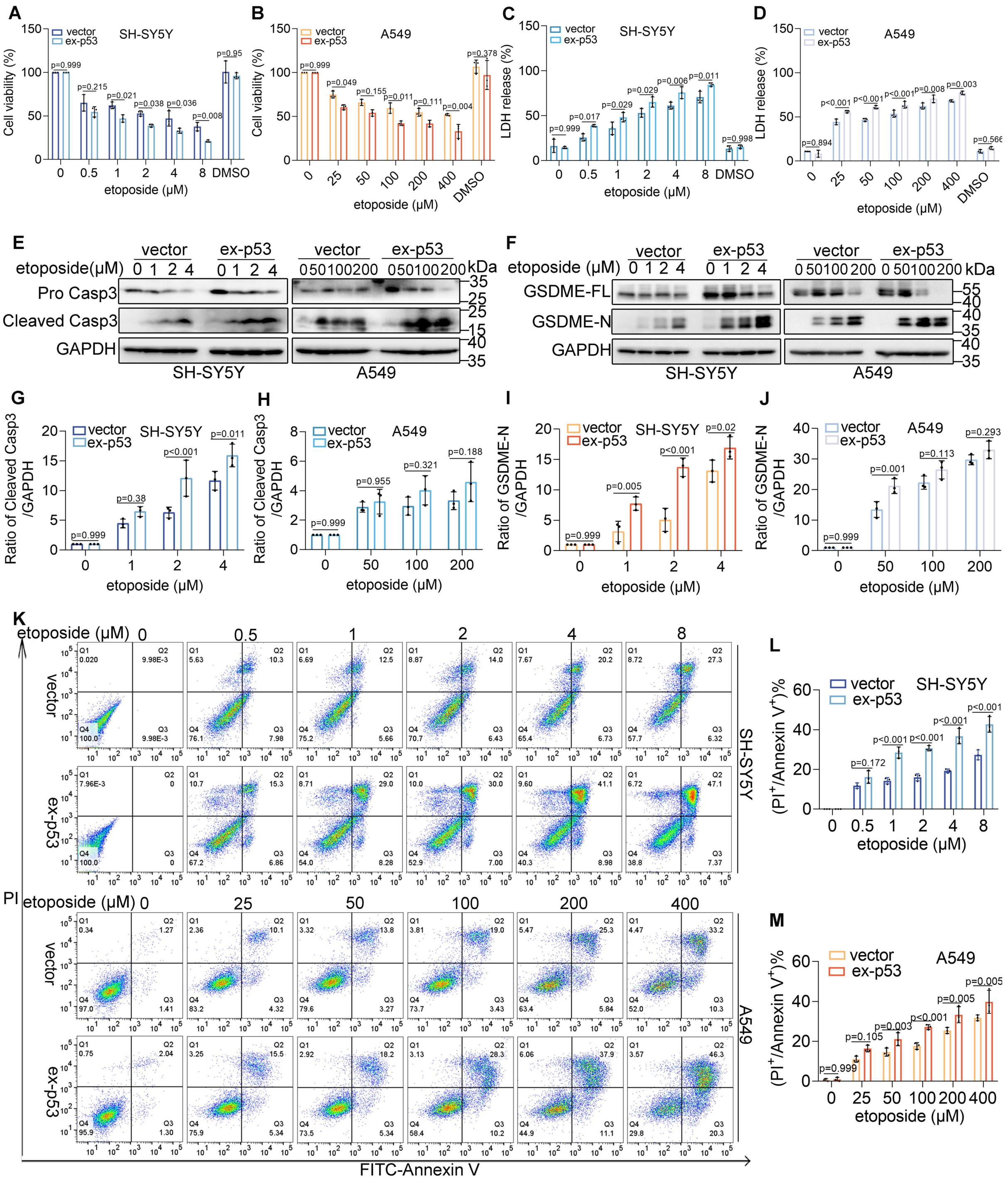

Figure S8. Biological functions of p53 in pyroptosis. **A-B** CCK8 was used to detect the cell viability of SH-SY5Y (A) and A549 (B) cells treated with etoposide in the control (vector) and overexpressed p53 groups (ex-p53). **C-D** LDH assay kit assessed the percentage of LDH release form SH-SY5Y (C) and A549 cells (D) treated with etoposide in the control (vector) and overexpressed p53 groups (ex-p53). **E-F** The control (vector) and overexpressed p53 groups (ex-p53) were treated with different concentrations of etoposide, and the activation of GSDME (E) and caspase-3 (F) were detected by western blotting. **G-J** The relative expression of GSDME and caspase-3. **K** The percentages of AnnexinV and PI double-positive cells

in the etoposide-treated control and overexpressed p53 groups (ex-p53) were measured by flow cytometry. **L-M** Quantification of the percentages of double positive cells. Data represent mean ± SD (n = 3) with significance determined by two-way ANOVA.

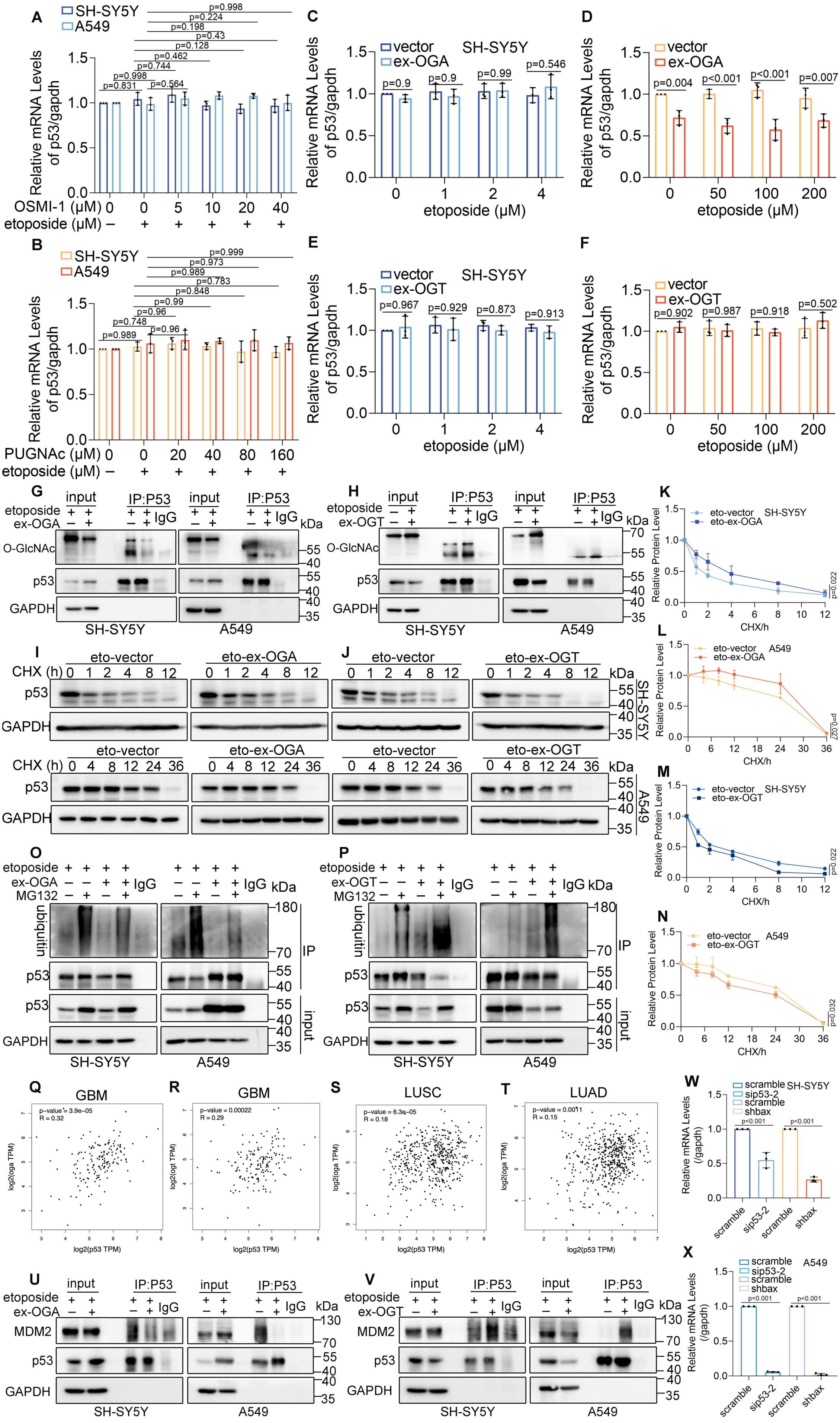
A
SH-SY5Y
A549
Relative mRNA Levels of p53/gapdh
OSMI-1 (μM) 0 0 5 10 20 40
etoposide (μM) − + + + + +
p=0.998
p=0.224
p=0.198
p=0.43
p=0.128
p=0.462
p=0.744
p=0.998
p=0.831
p=0.564
B
SH-SY5Y
A549
Relative mRNA Levels of p53/gapdh
PUGNAc (μM) 0 0 20 40 80 160
etoposide (μM) − + + + + +
p=0.999
p=0.973
p=0.989
p=0.783
p=0.848
p=0.99
p=0.96
p=0.748
p=0.989
p=0.96
C
vector
ex-OGA
SH-SY5Y
Relative mRNA Levels of p53/gapdh
p=0.9
p=0.9
p=0.99
p=0.546
etoposide (μM)
D
vector
ex-OGA
Relative mRNA Levels of p53/gapdh
p=0.004
p<0.001
p<0.001
p=0.007
etoposide (μM)
E
vector
ex-OGT
SH-SY5Y
Relative mRNA Levels of p53/gapdh
p=0.967
p=0.929
p=0.873
p=0.913
etoposide (μM)
F
vector
ex-OGT
Relative mRNA Levels of p53/gapdh
p=0.902
p=0.987
p=0.918
p=0.502
etoposide (μM)
G
input
IP:P53
etoposide
ex-OGA
IgG
kDa
O-GlcNAc
p53
GAPDH
SH-SY5Y
A549
H
input
IP:P53
etoposide
ex-OGT
IgG
kDa
O-GlcNAc
p53
GAPDH
SH-SY5Y
A549
K
eto-vector
eto-ex-OGA
SH-SY5Y
Relative Protein Level
CHX/h
p=0.022
L
eto-vector
eto-ex-OGA
A549
Relative Protein Level
CHX/h
p=0.027
I
eto-vector
eto-ex-OGA
CHX (h)
p53
GAPDH
J
eto-vector
eto-ex-OGT
kDa
SH-SY5Y
A549
M
eto-vector
eto-ex-OGT
SH-SY5Y
Relative Protein Level
CHX/h
p=0.022
N
eto-vector
eto-ex-OGT
A549
Relative Protein Level
CHX/h
p=0.032
O
etoposide
ex-OGA
MG132
IgG
kDa
ubiquitin
p53
GAPDH
IP
input
SH-SY5Y
A549
P
etoposide
ex-OGT
MG132
IgG
kDa
ubiquitin
p53
GAPDH
IP
input
SH-SY5Y
A549
Q
GBM
p-value = 3.9e-05
R = 0.32
log2(oga TPM)
log2(p53 TPM)
R
GBM
p-value = 0.00022
R = 0.29
log2(ogt TPM)
log2(p53 TPM)
S
LUSC
p-value = 6.3e-05
R = 0.18
log2(oga TPM)
log2(p53 TPM)
T
LUAD
p-value = 0.0011
R = 0.15
log2(oga TPM)
log2(p53 TPM)
W
scramble
sip53-2
scramble
shbax
SH-SY5Y
Relative mRNA Levels (/gapdh)
p<0.001
p<0.001
X
scramble
sip53-2
scramble
shbax
A549
Relative mRNA Levels (/gapdh)
p<0.001
p<0.001
U
input
IP:P53
etoposide
ex-OGA
IgG
kDa
MDM2
p53
GAPDH
SH-SY5Y
A549
V
input
IP:P53
etoposide
ex-OGT
IgG
kDa
MDM2
p53
GAPDH
SH-SY5Y
A549

Figure S9. O-GlcNAc modification of p53 reduces its stability. **A** RT-qPCR measured *p53* mRNA levels after osmi-1 treatment. **B** RT-qPCR assessed the expression of *p53* mRNA after pugNAc treatment. **C-D** RT-qPCR assessed the expression of *p53* mRNA in ex-OGA and vector cells. **E-F** RT-qPCR assessed the expression of *p53* mRNA in ex-OGT and vector cells. Data represent mean ± SD (n = 3) with significance determined by two-way ANOVA. **G** Immunoprecipitation detected the O-GlcNAcylation of p53 protein in vector and ex-OGA cells under etoposide treatment. **H** Immunoprecipitation detected the O-GlcNAcylation of p53 protein in vector and ex-OGT under etoposide treatment. **I** Western blotting detected the stability of p53 protein in vector and ex-OGA cells treated with etoposide. **J** Western blotting detected the stability of p53 protein in vector and ex-OGT cells treated with etoposide. **K-N** Quantification of (I-J). Mean ± SD, unpaired two-tailed t test, n = 3 biological replicates. **O** Western blotting detected the expression of p53 protein and its ubiquitination in vector and ex-OGA cells. **P** Western blotting assessed the expression of p53 protein and ubiquitination in vector and ex-OGT cells. **Q-T** GEPIA analyzed the correlation between p53 and O-GlcNAcylation-associated genes expression from the Cancer Genome Atlas (TCGA) GBM, LUSC and LUAD samples. **U** Vector and ex-OGA cells were treated with etoposide, the total levels of p53 and MDM2 were detected by western blotting, and the interaction between p53 and MDM2 was detected by co-immunoprecipitation; **V** Vector and ex-OGT cells were treated with etoposide, the total levels of p53 and MDM2 were detected by western blotting, and the interaction between p53 and MDM2 was detected by co-immunoprecipitation. **W-X** RT-qPCR was used to detect the mRNA levels of *p53* and *bax*. Data represent mean ± SD (n = 3) with significance determined by two-way ANOVA.

Table S1 Oligos for shRNA

Oligo	Sequences (5'-3')
sh-OGA-1	GCTTTGGACAGGTCCCAAAGT
sh-OGA-2	GCTCCAGTAATCTGGGATAAC
sh-OGA-3	CCACAAGAATGACAATCAAAT
sh-OGA-4	GCAGTTACTTGCTGATCTATT
sh-OGA-5	GGAGAGAGTCTTCAATAAATG
sh-OGA-6	GCATATGTGGTTATGCCTTGG
sh-OGT-1	GCCCTAAGTTTGAGTCCAA
sh-OGT-2	GCACGGCTGTGAAACTTAA
sh-OGT-3	TTTAGCACTCTGGCAATTAAA
sh-OGT-4	GCCCTAAGTTTGAGTCCAAAT
sh-OGT-5	GCTGAGCAGTATTCCGAGAAA
sh-Bax	ATCATCAGATGTGGTCTATAAGAT
sh-control	CAACAAGATGAAGAGCACCAA

Table S2 siRNA sequences

siRNA primer	Sequence (5’ - 3’)
sip53-1	CCCGGACGAUAUUGAACAATT
sip53-2	GCACAGAGGAAGAGAAUCUTT
sip53-3	CCACCAUCCACUACAACUATT
sicontrol (Negative control)	UUCUCCGAACGUGUCACGUTT

Table S3 Oligos for qPCR

Oligo	Sequence (5’-3’)
p53-F	GAGGTTGGCTCTGACTGTACC
p53-R	TCCGTCCCAGTAGATTACCAC
OGT-F	AGACGATGGCACAAACTTCC
OGT-R	ATCAGCTGCTTTTCCATTGC
OGA-F	TTCACTGAAGGCTAATGGCTCCCG
OGA-R	ATGTCACAGGCTCCGACCAAGT
GAPDH-F	ACAACTTTGGTATCGTGGAAGG
GAPDH-R	GCCATCACGCCACAGTTTC
